# A taxonomic revision of *Neoserica* (*sensu lato*): the species groups *N. lubrica*, *N. obscura*, and *N. silvestris* (Coleoptera, Scarabaeidae, Sericini)

**DOI:** 10.3897/zookeys.635.9915

**Published:** 2016-11-23

**Authors:** Wan-Gang Liu, Silvia Fabrizi, Ming Bai, Dirk Ahrens

**Affiliations:** 1 Institute of Earth and Environment, Chinese Academy of Sciences, Yanxiang Road 97#, Yanta District, Xi’an 710061 P.R. China; 2 Key Laboratory of Zoological Systematics and Evolution, Institute of Zoology, Chinese Academy of Sciences, Box 92, No. 1, Beichen West Road, Chaoyang District, Beijing, 100101, P.R. China; 3 Centre of Taxonomy and Evolutionary Research, Zoologisches Forschungsmuseum A. Koenig, Adenauerallee 160, 53113 Bonn, Germany

**Keywords:** Beetles, chafers, Neoserica, China, new species, new records

## Abstract

The species of the *Neoserica lubrica* Brenske, 1898, *Neoserica obscura* (Blanchard, 1850) and *Neoserica silvestris* Brenske, 1902 species groups are revised. The study resulted in the following new synonymies and combinations: *Neoserica obscura* (Blanchard, 1850) = *Microserica roeri* Frey, 1972, **syn. n.**, = *Maladera chinensis* (Arrow, 1946), **syn. n.**; *Neoserica hainana* (Brenske, 1898), **comb. n.**, and *Neoserica minor* (Arrow, 1946), **comb. n.** The known species are redescribed. The following nine new species are described from China: *Neoserica allobscura* Ahrens, Fabrizi & Liu, **sp. n.**, *Neoserica dongjiafenensis* Ahrens, Fabrizi & Liu, **sp. n.**, *Neoserica fugongensis* Ahrens, Fabrizi & Liu, **sp. n.**, *Neoserica mantillerii* Ahrens, Fabrizi & Liu, **sp. n.**, *Neoserica menglunensis* Ahrens, Fabrizi & Liu, **sp. n.**, *Neoserica pseudosilvestris* Ahrens, Fabrizi & Liu, **sp. n.**, *Neoserica sakoliana* Ahrens, Fabrizi & Liu, **sp. n.**, *Neoserica shuyongi* Ahrens, Fabrizi & Liu, **sp. n.**, and *Neoserica tahianensis* Ahrens, Fabrizi & Liu, **sp. n.** A key to the Sericini genera with multilamellate antenna, species groups of *Neoserica* of mainland Asia, and species of the species groups examined here are provided. Maps of the species distribution are provided, habitus and male genitalia are illustrated.

## Introduction

In the course of the revision of the species-rich genus *Neoserica* Brenske, 1894, of China a series of papers was published recently (Ahrens et al. 2014a–c, Liu et al. 2014a–c, 2015). In continuation of this work, here we present the results of the revision of the *Neoserica lubrica*, *Neoserica obscura* and *Neoserica silvestris* species groups.

As shown earlier (Ahrens 2003, 2004), *Neoserica* (*sensu lato*) comprises a polyphyletic mix of the larger species with multi-lamellate antenna (Liu et al. 2015) which require a revision of their nomenclature once their taxonomy, morphology, and phylogeny are better known. Apart from a number of new and interesting locality records, examined material also contained a number of new species which are described herein.

## Material and methods

The terminology and methods used for measurements, specimen dissection and genital preparation follow Ahrens (2004). Data from specimens examined are cited in the text with original label contents given in quotation marks, multiple labels are separated by a “/”. Descriptions and illustrations of the new taxa are based on the holotype if not otherwise stated, while the variation of specimens is given separately under “variation”. Remarks of the authors and comments are indicated in square brackets. Male genitalia were glued to a small pointed card and photographed in both lateral and dorsal view using a stereomicroscope Leica M125 with a Leica DC420C digital camera or a Zeiss AxioCam HRc digital camera mounted on a Zeiss Stereo Discovery.V20 stereomicroscope. With the Automontage software, a number of serial images were combined in order to obtain an entirely focused image. The resulting images were subsequently digitally edited in Artweaver (www.artweaver.de).

Abbreviations used in the text for the collection depositories are as follows:

BMNH
Natural History Museum, London, UK 


BPBM
 Bernice P. Bishop Museum, Honolulu, USA 


CN
 collection M. Nikodým, Prague, Czech Republic 


CP
 collection P. Pacholátko, Brno, Czech Republic 


CS
 collection G. Sabatinelli, Prevessin, France 


HBUM
Museum of Hebei University, Baoding, Hebei Province, China 


IZAS
Institute of Zoology, Chinese Academy of Sciences, Beijing, China 


MHNG
Muséum d’Histoire Naturelle, Genève, Switzerland 


MNHN
Museum national d’Histoire naturelle, Paris, France 


NHMW
 Naturhistorisches Museum Wien, Austria 


NHRS
Naturhistoriska Riksmuseet Stockholm, Sweden 


NMPC
National Museum Prague (Natural History), Prague, Czech Republic 


NWAFU
 Northwest A & F University, Yangling, Shaanxi Province, China 


SMFD
 Senckenbergmuseum, Frankfurt Main, Germany 


SNUC
 Shanghai Normal University, Department of Biology, China 


SYUG
 Sun Yat-Sen University, Guangzhou, China 


USNM
National Museum of Natural History, Washington D.C., U.S.A. 


ZFMK
 Zoologisches Forschungsmuseum A. Koenig, Bonn, Germany 


ZMHB
Museum für Naturkunde, Berlin, Germany 


## Results

### Key to the Sericini genera and *Neoserica* species groups with multi-lamellate antennal club (the key is so far suitable only for species known with both sexes):

**Table:** 

1	Hypomeron not carinate	***Tetraserica* Ahrens, 2004**
–	Hypomeron carinate	**2**
2	Antennal club in female composed of 3 antennomeres	**3**
–	Antennal club in female composed of more than 3 antennomeres	**16**
3	Posterior margin of metafemur serrate ventrally and dorsally	**4**
–	Posterior margin of metafemur smooth ventrally	**7**
4	Anterior angles of pronotum obsolete	**5**
–	Anterior angles of pronotum acute and moderately produced	**Neoserica (s.l.) calva group**
5	Dorsal surface nearly glabrous	***Gastroserica* Brenske, 1897**
–	Dorsal surface densely setose	**6**
6	Metatibia beside dorsal margin with a serrated longitudinal line or carina	***Neoserica* (s.str.) Brenske, 1894**
–	Metatibia beside dorsal margin without a serrated longitudinal line or carina	***Calloserica* Brenske, 1894**
7	Metatibia beside dorsal margin with a serrated longitudinal line or carina	**8**
–	Metatibia beside dorsal margin without a serrated longitudinal line or carina	**9**
8	Metatibia with one group of robust spines	***Lasioserica* Brenske, 1896**
–	Metatibia with two groups of robust spines	**Neoserica (s.l.) silvestris group**
9	Antennal club in males long and reflexed	***Anomalophylla* Reitter, 1887**
–	Antennal club in males short or moderately long and straight	**10**
10	Protibia bidentate	**11**
–	Protibia tridentate	***Trioserica* Moser, 1922**
11	Elytra bicolored, yellowish or reddish brown and black	**12**
–	Elytra unicolored	**13**
12	Parameres symmetrical	***Oxyserica* Brenske, 1900**
–	Parameres asymmetrical	***Microserica* Brenske, 1894**
13	Apex of metatibia shallowly truncate at interior apex near tarsal articulation	**14**
–	Apex of metatibia sharply truncate at interior apex near tarsal articulation	**15**
14	Dorsal surface yellowish brown to reddish brown, strongly and simply shiny	**Neoserica (s.l.) lubrica group**
–	Dorsal surface dull or iridescent shiny	**Neoserica (s.l.) vulpes group, other *Neoserica* (s.l.)**
15	Pronotum and elytra always nearly glabrous	***Sericania* Motschulsky, 1860** (see also couplet 21)
–	Pronotum and elytra always distinctly setose	***Gynaecoserica* Brenske, 1896**
16	Labrum without a transverse rim of very dense, short and robust setae	**17**
–	Labrum short, with a transverse rim of very dense, short and robust setae. Dorsal surface densely setose	**Neoserica (s.l.) pilosula group**
17	Metatibia slender and long	**19**
–	Metatibia short and wide	**18**
18	Body smaller 8.5 mm	**Neoserica (s.l.) obscura group**
–	Body larger 9 mm	**Neoserica (s.l.) uniformis group** & **Neoserica (s.l.) multifoliata group** (from Indochina)
19	Antennal club of males with 7 antennomeres	**20**
–	Antennal club of males with 6 or less antennomeres	**21**
20	Metafemur with a continuously serrated line adjacent to the anterior margin of metafemur. Protibia more or less distinctly tridentate	**Neoserica (s.l.) septemlamellata group**
–	Metafemur without a continuously serrated line adjacent to the anterior margin of metafemur. Protibia always distinctly bidentate	***Nepaloserica* Frey, 1965**
21	Basis of labroclypeus dull. Antennal club of males with 6 antennomeres	**22**
–	Antennal club of males with 5 or 4 antennomeres	**23**
22	Angle between basis of hypomeron and that of pronotum strongly rounded, angle between surfaces of hypomeron and pronotum basally blunt. Hypomeron basally strongly produced ventrally and transversely sulcate	***Lepidoserica* Nikolaev, 1979**
–	Angle between basis of hypomeron and that of pronotum sharp, angle between surfaces of hypomeron and pronotum sharp. Hypomeron basally not produced ventrally and not sulcate	**Neoserica (s.l.) abnormis group**
23	Apex of metatibia shallowly truncate at interior apex near tarsal articulation	**24**
–	Apex of metatibia deeply truncate at interior apex near tarsal articulation	***Sericania* Motschulsky, 1860** (see 14)
24	Body surface strongly shiny. Body smaller (5.7–6.6 mm)	**Neoserica (s.l.) speciosa group**
–	Body surface dull. Body larger (8 mm)	***Chrysoserica* Brenske, 1897**

### *Neoserica lubrica* group

**Diagnosis.** Body small (6–8 mm), oval, moderately convex; often unicoloured yellowish to reddish brown, entire dorsal surface strongly shiny and glabrous. Antenna with 10 antennomeres, yellow; antennal club of ♂ composed of 4–5 antennomeres, in ♀ of 3 antennomeres. Base of hypomeron carinate. Protibia bidentate. Metatibia at apex moderately sinuate near tarsal articulation. Metafemur without serrated line adjacent to anterior margin. Metatibia moderately wide, without serrated longitudinal line.

**Remarks.** The species group was based on *Neoserica lubrica* Brenske, 1898 (Ahrens 2004) to accommodate the species closely related to *Neoserica lubrica* (from Myanmar).

**Distribution.** Eastern Himalaya and northeastern India, southern China and Indochina.

#### Key to the Chinese species of the *Neoserica lubrica* group:

**Table:** 

1	Labrum without densely setose carina	**2**
–	Labrum with densely setose carina	***Neoserica menglunensis* Ahrens, Fabrizi & Liu, sp. n.**
2	Distal hook of left paramere nearly half as long as paramere itself	**3**
–	Distal hook of left paramere shorter than one quarter of length of paramere itself	***Neoserica dongjiafenensis* Ahrens, Fabrizi & Liu, sp. n.**
3	Distal hook of left paramere strongly curved and at apex bent backwards	***Neoserica mantillerii* Ahrens, Fabrizi & Liu, sp. n.**
–	Distal hook of left paramere moderately curved and at apex bent externally only	***Neoserica fugongensis* Ahrens, Fabrizi & Liu, sp. n.**

#### Neoserica (s.l.) fugongensis

Taxon classificationAnimaliaColeopteraScarabaeidae

Ahrens, Fabrizi & Liu sp. n.

http://zoobank.org/9E36767D-BF22-422D-AC35-0AC1E88ECF75

Figs 1A–D , 6

##### Type material examined.

Holotype: ♂ “China (Yunnan) Nujiang Lisu Aut. Pref., Salween side valley, 5 km S Fugong, road SS228, km 223 (creek bank, litter sifted) 8.VI.2007 leg. D. Wrase/ X-DA1554” (ZFMK).

##### Diagnosis.

The new species has the genitalia similar in shape to *Neoserica incompta* Ahrens & Fabrizi, 2009, but *Neoserica fugongensis* differs by the lens-shaped labrum and lacking the anterior fringe of dense setae on the labrum.

##### Description.

Body length: 6.7 mm, length of elytra: 4.5 mm, body width: 3.7 mm. Body oval, yellowish brown, dorsal surface strongly shiny and glabrous.

Labroclypeus subtrapezoidal, distinctly wider than long, widest at base, lateral margins nearly straight, convergent anteriorly, anterior angles strongly rounded, anterior margin very shallowly sinuate medially, margins moderately reflexed; surface convexly elevated at centre, shiny, finely and sparsely punctate, with a few single setae anteriorly; frontoclypeal suture distinctly incised, slightly elevated and weakly curved; smooth area anterior to eye weakly convex, approximately 1.5 times as wide as long; ocular canthus short and narrow (1/3 of ocular diameter), impunctate, with one terminal seta. Frons with fine and sparse punctures, with a few long erect setae beside eyes and on posterior half of frons. Eyes large, ratio diameter/ interocular width: 0.69. Antenna with ten antennomeres, club with five antennomeres and straight, slightly longer than remaining antennomeres combined. Mentum elevated and convex anteriorly. Labrum short, lens-shaped in anterior view, not produced medially, with shallow median sinuation and without densely setose anterior margin.

Pronotum moderately transverse, widest shortly behind middle, lateral margins evenly convex and weakly convergent towards base, more strongly convergent anteriorly; anterior angles distinctly produced and sharp, posterior angles blunt and weakly rounded at tip; anterior margin straight, with a fine complete marginal line; surface moderately densely and finely punctate, glabrous; lateral and anterior border sparsely setose; hypomeron distinctly carinate basally, not produced ventrally. Scutellum narrow, triangular, with fine, dense punctures, impunctate on basal midline, glabrous.

Elytra oval, widest at posterior third, striae finely impressed, finely and densely punctate, intervals nearly flat, with sparse, fine punctures concentrated along striae, glabrous; epipleural edge fine, ending at widely rounded external apical angle of elytra, epipleura densely setose, apical border with a fine fringe of microtrichomes (visible at 100× magnification).

Ventral surface shiny, finely and densely punctate, metasternum glabrous; metacoxa glabrous, with a few single setae laterally; abdominal sternites finely and densely punctate, with a transverse row of coarse punctures, each bearing a short robust seta. Mesosternum between mesocoxae as wide as the mesofemur. Ratio of length of metepisternum/ metacoxa: 1/ 1.55. Pygidium moderately convex and shiny, finely and sparsely punctate, without smooth midline, with a few long setae along apical margin.

Legs short; femora shiny, with two rudimentary longitudinal rows of setae, superficially and sparsely punctate, glabrous; metafemur with anterior margin acute, without serrated line behind anterior edge, posterior margin smooth ventrally in apical half only weakly widened, posterior margin smooth dorsally. Metatibia moderately wide and short, widest at middle, ratio of width/ length: 1/ 2.8; dorsal margin sharply carinate, with two groups of spines, basal group at one third, apical group at two thirds of metatibial length, basally with a few short single setae; lateral face weakly convex, finely and very sparsely punctate, smooth along the middle; ventral edge finely serrated, with three robust nearly equidistant setae; medial face smooth, apex interiorly near tarsal articulation bluntly truncate. Tarsomeres ventrally with sparse, short setae, smooth, neither laterally nor dorsally carinate; metatarsomeres with a strongly serrated ridge ventrally, smooth; first metatarsomere slightly shorter than following two tarsomeres combined and distinctly longer than dorsal tibial spur. Protibia moderately long, bidentate, distal tooth sharply pointed at apex; anterior claws symmetrical, basal tooth of inner claw sharply truncate at apex.

**Figure 1.**
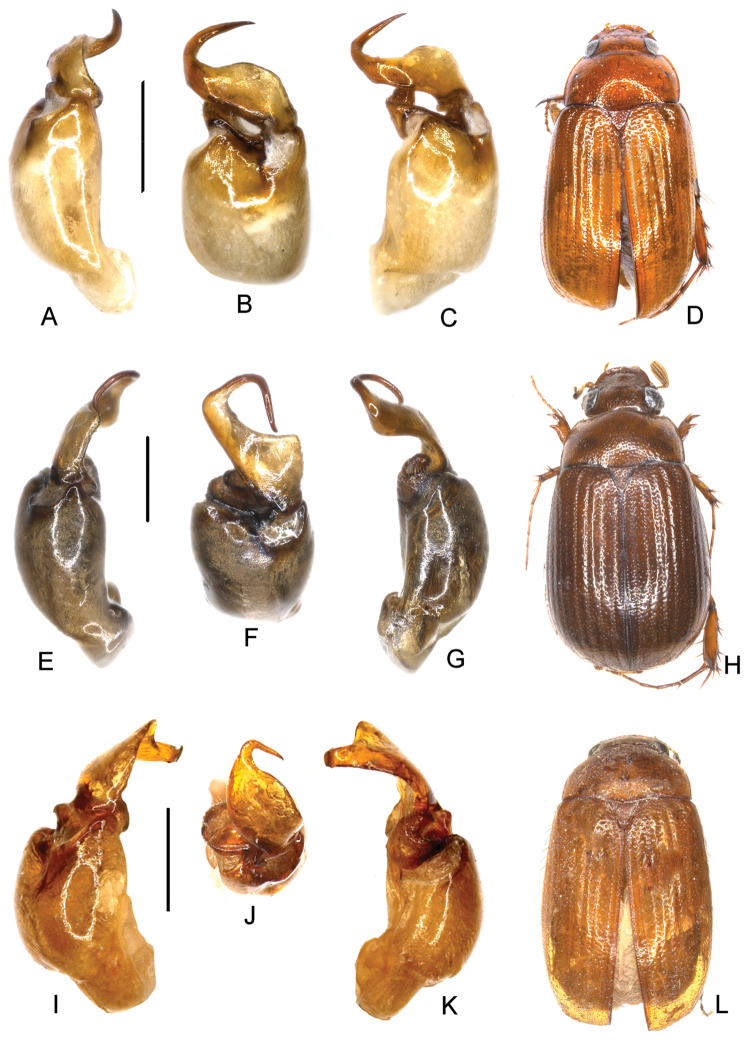
**A–D** *Neoserica fugongensis* Ahrens, Fabrizi & Liu, sp. n. (holotype) **E–H** *Neoserica mantillieri* Ahrens, Fabrizi & Liu, sp. n. (holotype) **I–L** *Neoserica dongjiafenensis* Ahrens, Fabrizi & Liu, sp. n. (holotype) **A, E** aedeagus, left side lateral view **C, G** aedeagus, right side lateral view **B, F** parameres, dorsal view **D, H** habitus. Scale bars: 0.5 mm. Habitus not to scale.

Aedeagus: Fig. 1A–C. Habitus: Fig. 1D. Female unknown.

##### Etymology.

This new species is named with reference to its type locality, Fugong.

#### Neoserica (s.l.) mantillerii

Taxon classificationAnimaliaColeopteraScarabaeidae

Ahrens, Fabrizi & Liu sp. n.

http://zoobank.org/83803BBD-4FB9-4691-B8A7-9DA3267CBCF9

Figs 1E–H , 6

##### Type material examined.

Holotype: ♂ “CHINE - Yunnan Tongbinguan 24°36'N, 97°35'E alt. 1180m/ Museum Paris 13.VI.2001 Deuve, Mantilleri, Rougerie & Tian leg.” (MNHN).

##### Diagnosis.

The new species is very similar to *Neoserica fugongensis* in the shape of the genitalia and in external appearance, but *Neoserica mantillierii* differs by the shape of the left paramere: it is longer and its external margin has a blunt angle in the middle, its apex is hook-like and strongly bent backwards.

##### Description.

Body length: 6.8 mm, length of elytra: 4.5 mm, body width: 3.7 mm. Body oval, yellowish brown, dorsal surface strongly shiny and glabrous.

Labroclypeus subtrapezoidal, distinctly wider than long, widest at base, lateral margins nearly straight, convergent anteriorly, anterior angles strongly rounded, anterior margin very shallowly sinuate medially, margins moderately reflexed; surface convexly elevated at centre, shiny, finely and sparsely punctate, with a few single setae anteriorly; frontoclypeal suture distinctly incised, slightly elevated and weakly curved; smooth area anterior to eye weakly convex, approximately 1.5 times as wide as long; ocular canthus short and narrow (1/3 of ocular diameter), impunctate, with one terminal seta. Frons with fine and sparse punctures, with a few long erect setae beside eyes and on posterior half of frons. Eyes large, ratio diameter/ interocular width: 0.69. Antenna with ten antennomeres, club with five antennomeres and straight, slightly longer than remaining antennomeres combined. Mentum elevated and convex anteriorly. Labrum short, lens-shaped in anterior view, not produced medially, with shallow median sinuation and without densely setose anterior margin.

Pronotum moderately transverse, widest shortly behind middle, lateral margins evenly convex and weakly convergent towards base, more strongly convergent anteriorly; anterior angles distinctly produced and sharp, posterior angles blunt and weakly rounded at tip; anterior margin weakly convex, with a fine complete marginal line; surface moderately densely and finely punctate, glabrous; lateral and anterior border sparsely setose; hypomeron distinctly carinate basally, not produced ventrally. Scutellum narrow, triangular, with fine, dense punctures, impunctate on basal midline, glabrous.

Elytra oval, widest at posterior third, striae finely impressed, finely and densely punctate, intervals nearly flat, with sparse, fine punctures concentrated along striae, glabrous; epipleural edge fine, ending at widely rounded external apical angle of elytra, epipleura densely setose, apical border with a fine fringe of microtrichomes (visible at 100× magnification).

Ventral surface shiny, finely and densely punctate, metasternum glabrous; metacoxa glabrous, with a few single setae laterally; abdominal sternites finely and densely punctate, with a transverse row of coarse punctures, each bearing a short robust seta. Mesosternum between mesocoxae as wide as the mesofemur. Ratio of length of metepisternum/ metacoxa: 1/ 1.42. Pygidium moderately convex and shiny, finely and sparsely punctate, without smooth midline, with a few long setae along apical margin.

Legs short; femora shiny, with two rudimentary longitudinal rows of setae, superficially and sparsely punctate, glabrous; metafemur with anterior margin acute, without serrated line behind anterior edge, posterior margin smooth ventrally in apical half only weakly widened, posterior margin smooth dorsally. Metatibia moderately wide and short, widest at middle, ratio of width/ length: 1/ 2.8, dorsal margin sharply carinate, with two groups of spines, basal group at one third, apical group at three quarters of metatibial length, basally with a few short single setae; lateral face weakly convex, finely and sparsely punctate, smooth along the middle; ventral edge finely serrated, with three robust nearly equidistant setae; medial face smooth, apex interiorly near tarsal articulation bluntly truncate. Tarsomeres ventrally with sparse, short setae, smooth, neither laterally nor dorsally carinate; metatarsomeres with a strongly serrated ridge ventrally, smooth; first metatarsomere slightly shorter than following two tarsomeres combined and distinctly longer than dorsal tibial spur. Protibia moderately long, bidentate, distal tooth sharply pointed at apex; anterior claws symmetrical, basal tooth of inner claw sharply truncate at apex.

Aedeagus: Fig. 1E–G. Habitus: Fig. 1H. Female unknown.

##### Etymology.

This new species is named after one of its collectors, Mr. Mantilleri, who provided us with a series of unidentified specimens from his expedition to China.

#### Neoserica (s.l.) dongjiafenensis

Taxon classificationAnimaliaColeopteraScarabaeidae

Ahrens, Fabrizi & Liu sp. n.

http://zoobank.org/355E6236-4249-4A86-8915-462111D2939F

Figs 1I–L , 6

##### Type material examined.

Holotype: ♂ “Dongjiafen, Jingdong, Yunnan, 16.VI.1956, leg. Zaguljaev” (IZAS). Paratype: 1 ♂ “Jingdong, Yunnan, 23.VI.1956, light trap, leg. Krshzhanovsknja” (ZFMK).

##### Diagnosis.

The new species is in shape of genitalia and in external appearance very similar to *Neoserica fugongensis* and *Neoserica mantillierii* but differs distinctly in the shape of the left paramere: the distal hook in *Neoserica dongjiafenensis* is much shorter compared to the total length of the paramere whose basal portion is nearly as wide as long and nearly lobiform.

##### Description.

Body length: 6.4 mm, length of elytra: 4.5 mm, body width: 3.5 mm. Body oval, yellowish brown, dorsal surface strongly shiny and glabrous.

Labroclypeus subtrapezoidal, distinctly wider than long, widest at base, lateral margins nearly straight, convergent anteriorly, anterior angles strongly rounded, anterior margin very shallowly sinuate medially, margins moderately reflexed; surface convexly elevated at centre, shiny, finely and sparsely punctate, with a few single setae anteriorly; frontoclypeal suture distinctly incised, slightly elevated and weakly curved; smooth area anterior to eye weakly convex, approximately 1.5 times as wide as long; ocular canthus short and narrow (1/3 of ocular diameter), impunctate, with one terminal seta. Frons with fine and sparse punctures, with a few long erect setae beside eyes and on posterior half of frons. Eyes moderately large, ratio diameter/ interocular width: 0.6. Antenna with ten antennomeres, club with five antennomeres and straight, as long as remaining antennomeres combined. Mentum elevated and convex anteriorly. Labrum short, lens-shaped in anterior view, not produced medially, with shallow median sinuation and without densely setose anterior margin.

Pronotum moderately transverse, widest at base, lateral margins evenly convex and weakly convergent anteriorly; anterior angles distinctly produced and sharp, posterior angles blunt and weakly rounded at tip; anterior margin weakly convex, with a fine complete marginal line; surface moderately densely and finely punctate, glabrous; lateral and anterior border sparsely setose; hypomeron distinctly carinate basally, not produced ventrally. Scutellum narrow, triangular, with fine, moderately dense punctures, impunctate on basal midline, glabrous.

Elytra oval, widest at posterior third, striae finely impressed, finely and densely punctate, intervals nearly flat, with sparse, fine punctures concentrated along striae, glabrous except a few long setae on penultimate lateral interval; epipleural edge fine, ending at widely rounded external apical angle of elytra, epipleura densely setose, apical border with a fine fringe of microtrichomes (visible at 100× magnification).

Ventral surface shiny, finely and densely punctate, metasternum glabrous; metacoxa glabrous, with a few single setae laterally; abdominal sternites finely and densely punctate, with a transverse row of coarse punctures, each bearing a short robust seta. Mesosternum between mesocoxae as wide as the mesofemur. Ratio of length of metepisternum/ metacoxa: 1/ 1.64. Pygidium moderately convex and shiny, finely and sparsely punctate, without smooth midline, with a few long setae along apical margin.

Legs short; femora shiny, with two rudimentary longitudinal rows of setae, superficially and sparsely punctate, glabrous; metafemur with anterior margin acute, without serrated line behind anterior edge, posterior margin smooth ventrally in apical half only weakly widened, posterior margin smooth dorsally. Metatibia moderately wide and short, widest at middle, ratio of width/ length: 1/ 2.8, dorsal margin sharply carinate, with two groups of spines, basal group at one third, apical group at three quarters of metatibial length, basally with a few short single setae; lateral face weakly convex, finely and sparsely punctate, smooth along the middle; ventral edge finely serrated, with three robust nearly equidistant setae; medial face smooth, apex interiorly near tarsal articulation bluntly truncate. Tarsomeres ventrally with sparse, short setae, smooth, neither laterally nor dorsally carinate; metatarsomeres with a strongly serrated ridge ventrally, smooth; first metatarsomere distinctly shorter than following two tarsomeres combined and slightly longer than dorsal tibial spur. Protibia moderately long, bidentate, distal tooth sharply pointed at apex; anterior claws symmetrical, basal tooth of inner claw sharply truncate at apex.

Aedeagus: Fig. 1I–K. Habitus: Fig. 1L. Female unknown.

##### Etymology.

The name of the new species is derived from the type locality, Dongjiafen.

##### Variation.

Body length: 5.3–6.4 mm, length of elytra: 4.2–4.5 mm, body width: 3.4–3.5 mm.

#### Neoserica (s.l.) menglunensis

Taxon classificationAnimaliaColeopteraScarabaeidae

Ahrens, Fabrizi & Liu sp. n.

http://zoobank.org/7F36A5E3-BACF-45F0-90B4-09671F612ACC

Figs 2A–D , 6

##### Type material examined.

Holotype: ♂ “[China] Menglun, Yunnan, 19.V.1991, leg. Wang Yinglun, Tian Binggang” (NWAFU). Paratype: 1 ♂ “[China] Guangxi, Shangsi Shiwandashan 2011-VII-7, 263m” (IZAS).

##### Diagnosis.

*Neoserica menglunensis* Ahrens, Fabrizi & Liu sp. n. differs from all other Chinese species of the *Neoserica lubrica* group by the presence of a transverse rim of dense setae on the anterior margin of labrum, and also by the shape of parameres: the left paramere is narrow and long (5 times as long as wide), and sharply pointed at its apex.

##### Description.

Body length: 5.5 mm, length of elytra: 4.0 mm, body width: 3.7 mm. Body oval, yellowish brown, dorsal surface strongly shiny and glabrous.

Labroclypeus short and subtrapezoidal, distinctly wider than long, widest at base, lateral margins nearly straight, convergent anteriorly, anterior angles moderately rounded, anterior margin broadly sinuate medially, margins moderately reflexed; surface nearly flat, shiny, finely and sparsely punctate, with a few single setae anteriorly; frontoclypeal suture distinctly incised, slightly elevated and weakly curved; smooth area anterior to eye weakly convex, approximately 1.5 times as wide as long; ocular canthus short and narrow (1/3 of ocular diameter), impunctate, with one terminal seta. Frons with fine and moderately dense punctures, with a few long erect setae beside eyes and behind frontoclypeal suture. Eyes moderately large, ratio diameter/ interocular width: 0.64. Antenna with ten antennomeres, club with five antennomeres and straight, slightly shorter than remaining antennomeres combined. Mentum elevated and convex anteriorly. Labrum short, nearly lens-shaped in anterior view, not produced medially, with shallow median sinuation and with a rim of dense setae near anterior margin.

Pronotum moderately transverse, widest at base, lateral margins in basal half nearly straight and moderately convergent to middle, evenly convex and weakly convergent anteriorly; anterior angles distinctly produced and sharp, posterior angles blunt and weakly rounded at tip; anterior margin weakly convex, with a fine complete marginal line; surface moderately densely and finely punctate, glabrous; lateral and anterior border sparsely setose; hypomeron distinctly carinate basally, not produced ventrally. Scutellum narrow, triangular, with fine, moderately dense punctures, impunctate on basal midline, glabrous.

Elytra oval, widest at posterior third, striae finely impressed, finely and densely punctate, intervals nearly flat, with sparse, fine punctures concentrated along striae, glabrous except a few long setae on penultimate lateral interval; epipleural edge fine, ending at widely rounded external apical angle of elytra, epipleura densely setose, apical border without a fine fringe of microtrichomes (visible at 100× magnification).

Ventral surface shiny, finely and densely punctate, metasternum glabrous; metacoxa glabrous, with a few single setae laterally; abdominal sternites finely and densely punctate, with a transverse row of coarse punctures, each bearing a short robust seta. Mesosternum between mesocoxae as wide as the mesofemur. Ratio of length of metepisternum/ metacoxa: 1/ 1.61. Pygidium moderately convex and moderately shiny, finely and densely punctate, without smooth midline, with a few long setae along apical margin.

Legs short; femora shiny, with two rudimentary longitudinal rows of setae, superficially and sparsely punctate, glabrous; metafemur with anterior margin acute, without serrated line behind anterior edge, posterior margin smooth ventrally in apical half only weakly widened, posterior margin smooth dorsally. Metatibia wide and short, widest at middle, ratio of width/ length: 1/ 2.4, dorsal margin sharply carinate, with two groups of spines, basal group at one third, apical group at three quarters of metatibial length, basally with a few short single setae; lateral face weakly convex, finely and sparsely punctate, smooth along the middle; ventral edge finely serrated, with three robust nearly equidistant setae; medial face smooth, apex interiorly near tarsal articulation bluntly truncate. Tarsomeres ventrally with sparse, short setae, smooth, neither laterally nor dorsally carinate; metatarsomeres with a strongly serrated ridge ventrally, smooth; first metatarsomere distinctly shorter than following two tarsomeres combined and only slightly longer than dorsal tibial spur. Protibia moderately long, bidentate, distal tooth sharply pointed at apex; anterior claws symmetrical, basal tooth of inner claw sharply truncate at apex.

**Figure 2.**
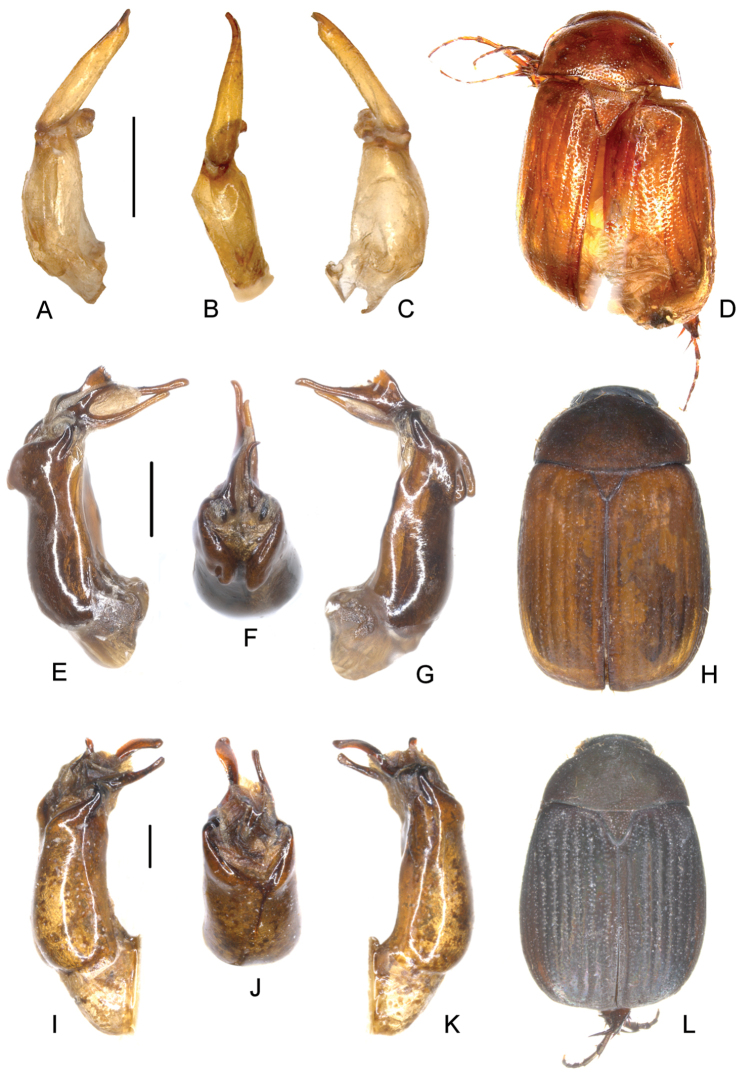
**A–D** *Neoserica menglungensis* Ahrens, Fabrizi & Liu, sp. n. (holotype) **E–H** *Neoserica obscura* (Blanchard) (holotype, *Microserica roeri* Frey) **I–L** *Neoserica allobscura* Ahrens, Fabrizi & Liu, sp. n. (holotype) **A, E** aedeagus, left side lateral view **C, G** aedeagus, right side lateral view **B, F** parameres, dorsal view **D, H** habitus. Scale bars: 0.5 mm. Habitus not to scale.

Aedeagus: Fig. 2A–C. Habitus: Fig. 2D. Female unknown.

##### Variation.

Body length: 5.4–5.5 mm, length of elytra: 3.9–4.0 mm, body width: 3.3–3.7 mm.

##### Etymology.

The new species is named after the type locality, Menglun.

### *Neoserica obscura* group

**Diagnosis.** Body small (6–8 mm), oval, strongly convex; often bicoloured black and reddish-brown, entire dorsal surface dull and nearly glabrous. Antenna with 10 antennomeres, dark; antennal club of ♂ composed of 4 antennomeres, in ♀ of 4 antennomeres, but club shorter than remaining antennomeres combined. Hypomeron basally carinate. Protibia bidentate. Metatibia at apex moderately sinuate close to tarsal articulation. Metafemur without serrated line adjacent to anterior margin. Metatibia moderately wide, without serrated longitudinal line.

**Remarks.** The species group was based on *Neoserica obscura* (Blanchard, 1850) proposed here to accommodate species closely related to *Neoserica obscura*.

**Distribution.** Eastern China and northern Indochina.

#### Key to the Chinese species of the *Neoserica obscura* group:

**Table:** 

1	Phallobase with a strong ventral lamina on the right side.	**2**
–	Phallobase without a ventral lamina.	**3**
2	Phallobase at apex with a pair of distinct dorsal protuberances	***Neoserica hainana***
–	Phallobase at apex without a pair of distinct dorsal protuberances	***Neoserica tahianensis* Ahrens, Fabrizi & Liu, sp. n.**
3	Phallobase at apex with a pair of distinct dorsal protuberances	***Neoserica obscura***
–	Phallobase at apex without a pair of distinct dorsal protuberances	**4**
4	Parameres distinctly less than half as long as phallobase	***Neoserica allobscura* Ahrens, Fabrizi & Liu, sp. n.**
–	Parameres half as long as phallobase	**5**
5	Right paramere nearly straight	***Neoserica sakoliana* Ahrens, Fabrizi & Liu, sp. n.**
–	Right paramere strongly bent ventrally behind middle	***Neoserica shuyongi* Ahrens, Fabrizi & Liu, sp. n.**

#### Neoserica (s.l.) obscura

Taxon classificationAnimaliaColeopteraScarabaeidae

(Blanchard, 1850)

Figs 2E–H , 6

Omaloplia obscura Blanchard, 1850: 79.
Neoserica obscura : Frey 1972a: 212, Ahrens 2006: 239, 2007: 26.
Microserica roeri Frey, 1972b: 171; Ahrens 2006: 239, 2007: 26; **syn. n.**
Aserica chinensis Arrow, 1946b: 268; Maladera (Aserica) chinensis: Ahrens 2006: 234, 2007: 19; **syn. n.**

##### Type material examined.

Lectotype (*Omaloplia obscura*, here designated): ♂ “Museum Paris Chine Gallery 5-46/ Omaloplia obscura Cat. Mus. China” (MNHN). Paralectotypes (*obscura*): 1 ♂ “Museum Paris Gallery 5-46/ Omaloplia obscura Blanch. ex Typis/ Brsk. I 98 vid” (MNHN), 1 ♂ “Museum Paris Chine Gallery 5-46” (MNHN). Syntype (*Aserica chinenesis*): 2 ♂♂ “China Kuliang 1923 S. F. Light” (BMNH), 1 ♂ “Foochow” (BMNH). Holotype (*Microserica roeri*): ♂ “Kuatun (2300 m) 27,40 n.Br. 117,49 ö.L. J. Klapperich 17.6.1938 (Fukien) /Type Microserica roeri n.sp. G. Frey 1971” (ZFMK). Paratypes (*Microserica roeri* FREY): 1 ♂ “Kuatun (2300 m) 27,40 n.Br. 117,49 ö.L. J. Klapperich 16.6.1938 (Fukien)/Paratype Microserica roeri n.sp. G. Frey 1971” (ZMHB), 3 ♂♂, 1 ♀ “Kuatun (2300 m) 27,40 n.Br. 117,49 ö.L. J. Klapperich 17.6.1938 (Fukien) /Paratype Microserica roeri n.sp. G. Frey 1971” (ZFMK), 1 ♀ “Kuatun (2300 m) 27,40 n.Br. 117,49 ö.L. J. Klapperich 15.6.1938 (Fukien) /Paratype Microserica roeri n. sp. G. Frey 1971” (ZFMK), 1 ex. (not sexed) “Kuatun (2300 m) 27,40 n.Br. 117,49 ö.L. J. Klapperich 25.5.1938 (Fukien)/ Microserica roeri m. G. Frey 1971/ Paratype Microserica roeri Frey, 1972 det. Ahrens 2016” (ZFMK), 4 ex. (not sexed) “Kuatun (2300 m) 27,40 n.Br. 117,49 ö.L. J. Klapperich 4.6.1938 (Fukien)/ Microserica roeri m. G. Frey 1971/ Paratype Microserica roeri Frey, 1972 det. Ahrens 2016” (ZFMK), 2 ex. (not sexed) “Kuatun (2300 m) 27,40 n.Br. 117,49 ö.L. J. Klapperich 12.6.1938 (Fukien)/ Microserica roeri m. G. Frey 1971/ Paratype Microserica roeri Frey, 1972 det. Ahrens 2016” (ZFMK), 15 ex. (not sexed) “Kuatun (2300 m) 27,40 n.Br. 117,49 ö.L. J.Klapperich 14.6.1938 (Fukien)/ Microserica roeri m. G. Frey 1971/ Paratype Microserica roeri Frey, 1972 det. Ahrens 2016” (ZFMK), 25 ex. (not sexed) “Kuatun (2300 m) 27,40 n.Br. 117,49 ö.L. J.Klapperich 15.6.1938 (Fukien)/ Microserica roeri m. G. Frey 1971/ Paratype Microserica roeri Frey, 1972 det. Ahrens 2016” (ZFMK), 27 ex. (not sexed) “Kuatun (2300 m) 27,40 n.Br. 117,49 ö.L. J.Klapperich 16.6.1938 (Fukien)/ Microserica roeri m. G. Frey 1971/ Paratype Microserica roeri Frey, 1972 det. Ahrens 2016” (ZFMK), 58 ex. (not sexed) “Kuatun (2300 m) 27,40 n.Br. 117,49 ö.L. J.Klapperich 17.6.1938 (Fukien)/ Microserica roeri m. G. Frey 1971/ Paratype Microserica roeri Frey, 1972 det. Ahrens 2016” (ZFMK), 9 ex. (not sexed) “Kuatun (2300 m) 27,40 n.Br. 117,49 ö.L. J.Klapperich 18.6.1938 (Fukien)/ Microserica roeri m. G. Frey 1971/ Paratype Microserica roeri Frey, 1972 det. Ahrens 2016” (ZFMK), 1 ex. (not sexed) “Kuatun (2300 m) 27,40 n.Br. 117,49 ö.L. J.Klapperich 20.6.1938 (Fukien)/ Microserica roeri m. G. Frey 1971/ Paratype Microserica roeri Frey, 1972 det. Ahrens 2016” (ZFMK), 1 ex. (not sexed) “Kuatun (2300 m) 27,40 n.Br. 117,49 ö.L. J.Klapperich 21.6.1938 (Fukien)/ Microserica roeri m. G. Frey 1971/ Paratype Microserica roeri Frey, 1972 det. Ahrens 2016” (ZFMK), 1 ex. (not sexed) “Kuatun (2300 m) 27,40 n.Br. 117,49 ö.L. J.Klapperich 9.7.1938 (Fukien)/ Microserica roeri m. G. Frey 1971/ Paratype Microserica roeri Frey, 1972 det. Ahrens 2016” (ZFMK), 1 ex. (not sexed) “Kuatun (2300 m) 27,40 n.Br. 117,49 ö.L. J.Klapperich 27.7.1938 (Fukien)/ Microserica roeri m. G. Frey 1971/ Paratype Microserica roeri Frey, 1972 det. Ahrens 2016” (ZFMK), 1 ex. (not sexed) “Kuatun (2300 m) 27,40 n.Br. 117,49 ö.L. J.Klapperich 8.8.1938 (Fukien)/ Microserica roeri m. G. Frey 1971/ Paratype Microserica roeri Frey, 1972 det. Ahrens 2016” (ZFMK), 4 ex. (not sexed) “Kuatun (2300 m) 27,40 n.Br. 117,49 ö.L. J.Klapperich 9.8.1938 (Fukien)/ Microserica roeri m. G. Frey 1971/ Paratype Microserica roeri Frey, 1972 det. Ahrens 2016” (ZFMK), 1 ex. (not sexed) “Kuatun (2300 m) 27,40 n.Br. 117,49 ö.L. J.Klapperich 22.8.1938 (Fukien)/ Microserica roeri m. G. Frey 1971/ Paratype Microserica roeri Frey, 1972 det. Ahrens 2016” (ZFMK).

##### Additional material examined.

8 ex. “China Fujian prov. Sangang env. 3.-5./.1991 M. Nikodým leg.” (ZFMK), 4 ex. “Chine 18.V.46 Kuatun, Fukien leg. Tschung-Sen” (MHNG), 18 ex. “Chine 1.VI.46 Kuatun, Fukien leg. Tschung-Sen” (MHNG), 4 ex. “Chine 4.VI.46 Kuatun, Fukien leg. Tschung-Sen” (MHNG), 3 ex. “Chine 8.VI.46 Kuatun, Fukien leg. Tschung-Sen” (MHNG), 12 ex. “Chine 10.VI.46 Kuatun, Fukien leg. Tschung-Sen” (MHNG), 6 ex. “Chine 12.VI.46 Kuatun, Fukien leg. Tschung-Sen” (MHNG), 57 ex. “Chine 15.VI.46 Kuatun, Fukien leg. Tschung-Sen” (MHNG), 78 ex. “Chine 20.VI.46 Kuatun, Fukien leg. Tschung-Sen” (MHNG), 14 ex. “Chine 29.VI.46 Kuatun, Fukien leg. Tschung-Sen” (MHNG), 37 ex. “Chine 28.VII.46 Kuatun, Fukien leg. Tschung-Sen” (MHNG), 6 ex. “Chine 4.VII.46 Kuatun, Fukien leg. Tschung-Sen” (MHNG), 24 ex. “Chine 6.VII.46 Kuatun, Fukien leg. Tschung-Sen” (MHNG), 24 ex. “Chine 7.VII.46 Kuatun, Fukien leg. Tschung-Sen” (MHNG), 36 ex. “Chine 14.VII.46 Kuatun, Fukien leg. Tschung-Sen” (MHNG), 18 ex. “Chine 22.VII.46 Kuatun, Fukien leg. Tschung-Sen” (MHNG), 3 ex. “Chine 14.VIII.46 Kuatun, Fukien leg. Tschung-Sen” (MHNG), 2 ex. “Reg. de Luc-Nam (Tonkin) L. Blaise/ Museum Paris (Coll. Ph. Francois) Coll. L. Bedel 1922” (MNHN), 4 ex. “Museum Paris Kouy-Tcheou P. Cavalerie 1910” (MNHN), 2 ex. “Museum Paris Kouy-Tcheou Reg. de Pin-Fa, P. Cavalerie 1908” (MNHN), 2 ex. “Museum Paris Kouy-Tcheou Kouy-Yang, P.P. Cavalerie et Fortunat 1906” (MNHN), 6 ex. “Hangchow 6-5-24/ J. F. Illingworth” (BPBM), 2 ex. “Kwangtung, S.C. Chukiang Lungtaushan 17.VI.1947/ J.L. Gressitt Collector” (BPBM), 1 ex. “S. China Kwangtung Loh-chang Dist. 1947/ J.L. Gressitt Colletor Bishop Museum” (BPBM), 3 ex. “S. China: Kwangtung Tsin-leong Shan 5.VI.1936/ L. &. M. Gressitt Collectors Bishop Museum” (BPBM), 1 ex. “S. China NE Kwangtung Yim-na Shan 10-15.VI.36/ J. L. Gressitt Collector Bishop Museum” (BPBM), 1 ex. “Fukien, S. China Kienyang: Nwangkeng 6.VI.42 T. C. Maa” (BPBM), 1 ex. “China: Taipe‘ v.1925/ D. T. Fullaway, Coll. Bishop Museum Acc. +1986.189” (BPBM), 2 ex. “Hong Kong: Hong Kong Island IV.1958/ N.L.H. Krauss Collector Bishop” (BPBM), 1 ex. “Fukien, S. China Shaowu: Tachulan 1000 m. 22.VII.42/ T. C. Maa Collector Bishop Mus.” (BPBM), 2 ex. “Fukien, S. China Kienyang City 1941-23.VI. Maa” (BPBM), 1 ex. “Fukien, S. China Kienyang: Kwang-keng to Tachulan 1943. Maa” (BPBM), 1 ex. “Fukien, S. China Shaowu: Tachulan 1000 m. T. Maa/ 1.VII.42” (BPBM), 1 ex. “Fukien, S. China Shaowu: Tachulan 1000 m. T. Maa/ 7.V.42” (BPBM), 2 ex. “Fukien, S. China Changting City 1940 3.VI. Maa” (BPBM), 1 ex. “Fukien, S. China Changting: Hotien 24.VII.1940/ T.C. Maa Collector Bishop” (BPBM), 1 ex. “Fukien, S. China Shahsien 15.VII.1940 T. Maa” (BPBM), 2 ex. “Kuatun (2300 m) 27,40 n.Br. 117,49 ö.L. J.Klapperich 17.6.1938 (Fukien)/ Neoserica obscura Bl. det G. Frey 1967/68” (CF), 3 ex. “Kiangsi, S. China Taiauhong, S. of Sungwu, 540 m VII-5-36 Gressitt” (BPBM), 1 ex. “Fukien, S. China Chungan Bohea Hills 12.VI.1941 T.C. Maa” (BPBM), 1 ex. “Foochow July‘24/ J.F. Illingworth” (BPBM), 2 ex. “Kiangsi, SE China Hong Shan 1000 m VI-25-36, Gressitt” (BPBM), 1 ex. “China, W Jiangxi Jinggang Shan-Ciping 2-14.VI.1994 E. Jendek & O. Sausa leg./ CS 18” (CP), 7 ex. “China, W Jiangxi Jinggang Shan Ciping env. 2-14.VI.1994” (NHMW), 46 ex. “China Hunan SE Ling Xian env. 26.31N 113.44E 15-18.VI.1994” (NHMW), 2 ex. “China Hunan S Chenzhou env. 25.49N 112.59E 19-21.VI.1994” (NHMW), 1 ex. “China Hunan SE Guidong env. 26.04N 113.56E 26-31.V.1994” (NHMW), 3 ex. “China Schf.” (ZMHB), 5 ex. “ China Canton” (ZMHB), 1 ex. “ Kiautschou China” (ZMHB), 1 ex. “China Canton V.-VII.11 Mell S.V.” (ZMHB), 3 ex. “China Tsha-jiu-san VII-IX.10 Mell S.V.” (ZMHB), 1 ex. “Prov.Fo-Kien (China)” (ZMHB), 1 ex. “Fockien Donckier” (ZMHB), 5 ex. “Museum Paris Chekiang Hang Tcheou A. Pichon 1925” (MNHN), 1 ex. “China, N Fujian, 8.-25,V. Wuyi Shan mts. ~10km W Xingcun pitfall traps, 27.65N 117.85E Jaroslav Turna leg., 2005” (ZFMK), 8 ex. “Kuatun (2300m) 27,40n.Br. 117,40o.L. J. Klapperich 17.6. 1938 (Fukien)/ ex. Coll. V. Balthasar National Museum Prague, Czech Republic” (NMPC), 2 ex. “Kuatun (2300m) 27,40n.Br. 117,40o.L. J. Klapperich 14.6. 1938 (Fukien)/ ex. Coll. V. Balthasar National Museum Prague, Czech Republic” (NMPC), 2 ex. “Kuatun (2300m) 27,40n.Br. 117,40o.L. J. Klapperich 16.6. 1938 (Fukien)/ ex. Coll. V. Balthasar National Museum Prague, Czech Republic” (NMPC), 1 ex. “Kuatun (2300m) 27,40n.Br. 117,40o.L. J. Klapperich 18.8. 1938 (Fukien)/ ex. Coll. V. Balthasar National Museum Prague, Czech Republic” (NMPC), 2 ex. “Kuatun (2300m) 27,40n.Br. 117,40o.L. J. Klapperich 25.5. 1938 (Fukien)/ ex. Coll. V. Balthasar National Museum Prague, Czech Republic” (NMPC), 5 ex. “Kuatun Fukien China 18.6.46 (Tschung Sen.)/ ex. Coll. V. Balthasar National Museum Prague, Czech Republic” (NMPC), 1 ex. “Kuatun Fukien China 15.6.46 (Tschung Sen.)/ ex. Coll. V. Balthasar National Museum Prague, Czech Republic” (NMPC), 1 ♂ “China: E Guizhou prov.; Fodingshan; Ganshi; 25km of Shiguian; 1300m; BOLM lgt.; 5.-9.vi.1997” (CP), 3 ex. “Datchulau China ‘39 TH Cheng/ July 39” (USNM), 2 ex. “Hangchow VI-22 1927 Coll. C.Y. Wong” (USNM), 3 ex. “Hangchow VI-23 1927 Coll. C.Y. Wong” (USNM), 1 ex. “Hangchow VI-24 1927 Coll. C.Y. Wong” (USNM), 1 ex. “Foochow China VII- 18 2519 Coll. C.C. Wee” (USNM), 1 ex. “Hangchow China./ VII-6 to VIII-17-1926 Coll. H.A. Jaynes” (USNM), 1 ex. “Hangchow China./ V-10-1926 Coll. H.A. Jaynes” (USNM), 1 ex. “Hangchow IX-20-1924/ J.F. Illingwerth Ex flower” (USNM), 1 ex. “Zakow China VII-16-1924” (USNM), 1 ex. “Zakow China./ III-24 to IV-15-1926 Coll. H.A. Jaynes” (USNM), 1 ex. “Foochow China CI-1928/ F.C. Hadden Collector” (USNM), 2 ex. “Chekiang China/ OL Cartwright collection 1959” (USNM), 1 ♂ “Guangdong, 9,10.VII.1965” (IZAS), 1 ♂ “Xiadao, Nanping, Fujian, 27.V.1963, leg. Zhang Youwei” (IZAS), 2 ♂♂, 1 ♀ “Hujiang, Lianxian County, Guangdong, 18.VI.1965, leg. Zhang Youwei” (IZAS), 1 ♂ “Guangdong, 7.VII.1965” (IZAS), 1 ♂ “Jiuniutang, Mao’ershan, Guangxi, 13.VII.1985, 1100m, leg. Liao Subai” (IZAS), 1 ♂ “Chong’an Xingcun, Fujian, 10.VII.1960, 200m, leg. Jiang Shengqiao” (IZAS), 1 ♂ “Xiangshui, Boluo, Guangdong, 31.V.1965, leg. Zhang Youwei” (IZAS), 1 ♂ “Guanping, Tongmuguan, Chong’an, Fujian, 13.VIII.1960, 900-1000m, leg. Jiang Shengqiao” (IZAS), 4 ex. “China: Zhejiang, Longquan City, 30.VI.1979” (SNUC).

##### Redescription.

Body length: 5.6 mm, length of elytra: 3.9 mm, body width: 3.6 mm. Body short-oval, black, elytra reddish brown, dorsal surface except anterior labroclypeus dull, pronotum and elytra glabrous.

Labroclypeus subtrapezoidal, distinctly wider than long, widest at base, lateral margins weakly convex, convergent anteriorly; anterior angles strongly rounded; anterior margin shallowly sinuate medially, margins moderately reflexed; surface weakly convex, shiny, base dull, coarsely and densely punctate, with numerous erect setae; frontoclypeal suture indistinctly incised, vanishing under dull toment; smooth area in front of eye convex, nearly as long as wide; ocular canthus short and triangular (1/3 of ocular diameter), sparsely punctate, with one or more terminal setae. Frons with fine and moderately dense punctures, with a few long erect setae beside eyes and behind frontoclypeal suture. Eyes small, ratio diameter/ interocular width: 0.41. Antenna with ten antennomeres, club (♂) with four antennomeres and straight, as long as remaining antennomeres combined. Mentum convexly elevated and flattened anteriorly.

Pronotum transverse, widest at base, lateral margins in basal half nearly straight and moderately convergent to middle, evenly convex and weakly convergent anteriorly; anterior angles distinctly produced and sharp, posterior angles blunt and weakly rounded at tip; anterior margin straight, with a fine complete marginal line; surface densely and finely punctate, glabrous, with minute setae in punctures (100× magnification); lateral border densely setose; hypomeron distinctly carinate basally, not produced ventrally. Scutellum triangular, with fine, dense punctures, glabrous.

Elytra short-oval, widest shortly behind middle, striae finely impressed, finely and densely punctate, intervals weakly convex, with sparse, fine punctures concentrated along striae, glabrous except a few single, short setae on odd intervals; epipleural edge robust, ending at nearly blunt external apical angle of elytra, epipleura densely setose; apical border without a fine fringe of microtrichomes (visible at 100× magnification).

Ventral surface dull, finely and densely punctate; metasternum nearly glabrous except a few long robust setae on disc, punctures with minute setae (100× magnification); metacoxa glabrous, with a few single setae laterally; abdominal sternites finely and densely punctate, with a transverse row of coarse punctures, each bearing a short robust seta, last sternite half as long as penultimate one. Mesosternum between mesocoxae as wide as the mesofemur, with a semi-circular ridge bearing long setae. Ratio of length of metepisternum/ metacoxa: 1/ 1.9. Pygidium dull, moderately convex, finely and densely punctate, without smooth midline, with a few long setae along apical margin.

Legs short; femora moderately shiny, with two rudimentary longitudinal rows of setae, finely and sparsely punctate, glabrous; metafemur with anterior margin acute, without serrated line behind anterior edge, posterior margin smooth ventrally, in apical half only weakly widened, posterior margin smooth dorsally. Metatibia wide and short, widest at middle, ratio of width/ length: 1/ 2.7; dorsal margin sharply carinate, with two groups of spines, basal group at one third, apical group at three quarters of metatibial length, basally with a few short single setae; lateral face weakly convex, finely and sparsely punctate, smooth along middle; ventral edge finely serrated, with three robust nearly equidistant setae; medial face smooth, apex interiorly near tarsal articulation bluntly truncate and slightly concavely sinuate. Tarsomeres ventrally with sparse, short setae, smooth, neither laterally nor dorsally carinate; metatarsomeres with a strongly serrated ridge ventrally, glabrous; first metatarsomere as long as following two tarsomeres combined and slightly longer than dorsal tibial spur. Protibia short, bidentate, distal tooth sharply pointed at apex; anterior claws symmetrical, basal tooth of inner claw sharply truncate at apex.

Aedeagus: Fig. 2E–G. Habitus: Fig. 2H.

##### Variation.

The colour may vary from being totally black to reddish brown. Female: antennal club also composed of 4 antennomeres, however, the club is slightly shorter than in males and the first joint of the club is slightly shorter than the club; pygidium moderately convex, at middle strongly shiny and finely punctate.

##### Remarks.

The parameres of the lectotype of *Neoserica obscura* (Blanchard) are virtually identical in the shape with those of *Microserica roeri* Frey and *Aserica chinensis* Arrow. The latter two names are consequently proposed here as junior synonyms of *Neoserica obscura*.

#### Neoserica (s.l.) allobscura

Taxon classificationAnimaliaColeopteraScarabaeidae

Ahrens, Fabrizi & Liu sp. n.

http://zoobank.org/04D24B11-EEC3-496B-AFCB-2E9311F9DD97

Fig. 2I–L

##### Type material examined.

Holotype: ♂ “China coll. Chev./ obscura Bl. Mit cotype vergl 4.I.98./ obscura Bl./coll. Brenske” (ZMHB).

##### Diagnosis.

*Neoserica allobscura* Ahrens, Fabrizi & Liu, sp. n. is in external appearance and genital morphology very similar to *Neoserica obscura*. *Neoserica allobscura* differs by the less distinct pair of protuberances on the dorsoapical phallobase and the shape of the parameres: the right paramere is strongly curved in the middle and its basal lobe is longer than the rudimentary one of *Neoserica obscura*; the dorsal lobe of the left paramere is displaced more basally and bent interiorly, while in *Neoserica obscura* it is directly above the ventral lobe of the left paramere.

##### Description.

Body length: 6.9 mm, length of elytra: 3.9 mm, body width: 3.6 mm. Body short-oval, dark brown, elytra black, dorsal surface except anterior labroclypeus dull, pronotum and elytra glabrous.

Labroclypeus subtrapezoidal, distinctly wider than long, widest at base, lateral margins weakly convex, convergent anteriorly; anterior angles strongly rounded; anterior margin shallowly sinuate medially, margins moderately reflexed; surface weakly convex, shiny, base dull, densely punctate, coarse punctures mixed with minute ones, with numerous erect setae; frontoclypeal suture indistinctly incised, weakly curved medially; smooth area in front of eye convex, nearly as long as wide; ocular canthus short and triangular (1/3 of ocular diameter), sparsely punctate, with one or more terminal setae. Frons with fine and moderately dense punctures, with a few long erect setae beside eyes and behind frontoclypeal suture. Eyes small, ratio diameter/ interocular width: 0.42. Antenna with ten antennomeres, club (♂) with four antennomeres and straight, as long as remaining antennomeres combined. Mentum convexly elevated and flattened anteriorly.

Pronotum transverse, widest at base, lateral margins evenly convex and moderately convergent anteriorly; anterior angles distinctly produced and sharp, posterior angles blunt and weakly rounded at tip; anterior margin straight, with a fine complete marginal line; surface densely and finely punctate, glabrous, with minute setae in punctures (100× magnification); lateral border densely setose; hypomeron distinctly carinate basally, not produced ventrally. Scutellum triangular, with fine, dense punctures, glabrous.

Elytra short-oval, widest shortly behind middle, striae finely impressed, finely and densely punctate, intervals weakly convex, with sparse, fine punctures concentrated along striae, glabrous; epipleural edge robust, ending at nearly blunt external apical angle of elytra, epipleura densely setose; apical border without a fine fringe of microtrichomes (visible at 100× magnification).

Ventral surface dull, finely and densely punctate; metasternum nearly glabrous except a few long robust setae on disc, punctures with minute setae (100× magnification); metacoxa glabrous, with a few single setae laterally; abdominal sternites finely and densely punctate, with a transverse row of coarse punctures, each bearing a short robust seta, last sternite half as long as penultimate one. Mesosternum between mesocoxae as wide as the mesofemur, with a semi-circular ridge bearing long setae. Ratio of length of metepisternum/ metacoxa: 1/ 2.2. Pygidium dull, moderately convex, coarsely and densely punctate, without smooth midline, with a few long setae along apical margin.

Legs short; femora moderately shiny, with two rudimentary longitudinal rows of setae, finely and sparsely punctate, glabrous; metafemur with anterior margin acute, without serrated line behind anterior edge, posterior margin smooth ventrally, in apical half only weakly widened, posterior margin smooth dorsally. Metatibia wide and short, widest at middle, ratio of width/ length: 1/ 2.7; dorsal margin sharply carinate, with two groups of spines, basal group at one third, apical group at three quarters of metatibial length, basally with a few short single setae; lateral face weakly convex, finely and sparsely punctate, smooth along middle; ventral edge finely serrated, with three robust nearly equidistant setae; medial face smooth, apex interiorly near tarsal articulation bluntly truncate and slightly concavely sinuate. Tarsomeres ventrally with sparse, short setae, smooth, neither laterally nor dorsally carinate; metatarsomeres with a strongly serrated ridge ventrally, glabrous; first metatarsomere as long as following two tarsomeres combined and as long as dorsal tibial spur. Protibia short, bidentate, distal tooth sharply pointed at apex; anterior claws symmetrical, basal tooth of inner claw sharply truncate at apex.

Aedeagus: Fig. 2I–K. Habitus: Fig. 2L. Female unknown.

##### Etymology.

The name of the new species is derived from the Greek prefix “allo-” (other) and the Latin adjective “obscurus” (dark) with reference to the name and the similarity to *Neoserica obscura*.

#### Neoserica (s.l.) hainana

Taxon classificationAnimaliaColeopteraScarabaeidae

(Brenske, 1898) comb. n.

Figs 3A–D , 6

Microserica hainana Brenske, 1898: 216.

##### Type material examined.

Lectotype (here designated): ♂ “Hainan Schmack/ Serica hainana var. type Brsk./ Coll. v. Schönfeldt” (SMFD). Paralectotypes: 4 ♂♂, 3 ♀♀ “Hainan Schmack/ Coll. v. Schönfeldt” (SMFD), 1 ♂ “hainana var. type/ Coll. v. Schönfeldt” (SMFD), 1 ♂ “Hainan Schmack/ Serica hainana type Brsk./ Coll. v. Schönfeldt/ hainana Brske” (SMFD), 1 ♀ “Hainan v.Schönfeldt/ Serica hainana type Brsk.” (ZMHB), 1 ♂ “19./ Hainan Schmack/ Serica hainana var. type Brsk./ Coll. v. Schönfeldt” (SMFD).

##### Additional material examined.

1 ♂ “Qiongzhong, Hainan, Guangdong, 17.VII.1960, 400m, leg. Zhang Xuezhong” (IZAS), 1 ♂, 1 ♀ “Bawangzhen, Changjiang, Hainan, 5-7.VI.2008, leg. Ba Yibin, Lang Juntong” (HBUM), 3 ♂♂ “Bawangzhen, Changjiang, Hainan, 5-7.VI.2008, leg. Ba Yibin, Lang Juntong” (HBUM).

##### Redescription.

Body length: 6.8 mm, length of elytra: 4.8 mm, body width: 4.2 mm. Body short-oval, black, elytra reddish brown, dorsal surface except anterior labroclypeus dull, pronotum and elytra glabrous.

Labroclypeus subtrapezoidal, distinctly wider than long, widest at base, lateral margins weakly convex, convergent anteriorly; anterior angles strongly rounded; anterior margin shallowly sinuate medially, margins moderately reflexed; surface weakly convex, shiny, base dull, coarsely and densely punctate, with numerous erect setae; frontoclypeal suture indistinctly incised, nearly vanishing under dull toment; smooth area in front of eye convex, nearly as long as wide; ocular canthus short and triangular (1/3 of ocular diameter), sparsely punctate, terminal setae in lectotype lacking. Frons with fine and moderately dense punctures, without erect setae. Eyes small, ratio diameter/ interocular width: 0.4. Antenna with ten antennomeres, yellowish, club (♂) with four antennomeres and straight, as long as remaining antennomeres combined. Mentum convexly elevated and flattened anteriorly.

Pronotum transverse, widest shortly before base, lateral margins in basal half nearly straight and moderately convergent to middle, evenly convex and weakly convergent anteriorly; anterior angles distinctly produced and sharp, posterior angles blunt and weakly rounded at tip; anterior margin straight, with a fine complete marginal line; surface densely and finely punctate, glabrous, with minute setae in punctures (100× magnification); lateral border densely setose; hypomeron distinctly carinate basally, not produced ventrally. Scutellum triangular, with fine, dense punctures, glabrous.

Elytra short-oval, widest shortly behind middle, striae finely impressed, finely and densely punctate, intervals weakly convex, with sparse, fine punctures concentrated along striae, glabrous except a few single, short setae on odd intervals; epipleural edge robust, ending at nearly blunt external apical angle of elytra, epipleura densely setose; apical border without a fine fringe of microtrichomes (visible at 100× magnification).

Ventral surface dull, finely and densely punctate; metasternum nearly glabrous except a few long robust setae on disc, punctures with minute setae (100× magnification); metacoxa glabrous, with a few single setae laterally; abdominal sternites finely and densely punctate, with a transverse row of coarse punctures, each bearing a short robust seta, last sternite half as long as penultimate one. Mesosternum between mesocoxae as wide as the mesofemur, with a semi-circular ridge bearing long setae. Ratio of length of metepisternum/ metacoxa: 1/ 2.0. Pygidium dull, moderately convex, finely and densely punctate, without smooth midline, with a few long setae along apical margin.

Legs short; femora moderately shiny, with two rudimentary longitudinal rows of setae, finely and sparsely punctate, glabrous; metafemur with anterior margin acute, without serrated line behind anterior edge, posterior margin smooth ventrally, in apical half only weakly widened, posterior margin smooth dorsally. Metatibia wide and short, widest at middle, ratio of width/ length: 1/ 2.8; dorsal margin sharply carinate, with two groups of spines, basal group at one third, apical group at three quarters of metatibial length, basally with a few short single setae; lateral face weakly convex, finely and sparsely punctate, smooth along middle; ventral edge finely serrated, with three robust nearly equidistant setae; medial face smooth, apex interiorly near tarsal articulation bluntly truncate and slightly concavely sinuate. Tarsomeres ventrally with sparse, short setae, smooth, neither laterally nor dorsally carinate; metatarsomeres with a strongly serrated ridge ventrally, glabrous; first metatarsomere slightly shorter than following two tarsomeres combined and slightly longer than dorsal tibial spur. Protibia short, bidentate, distal tooth sharply pointed at apex; anterior claws symmetrical, basal tooth of inner claw sharply truncate at apex.

**Figure 3.**
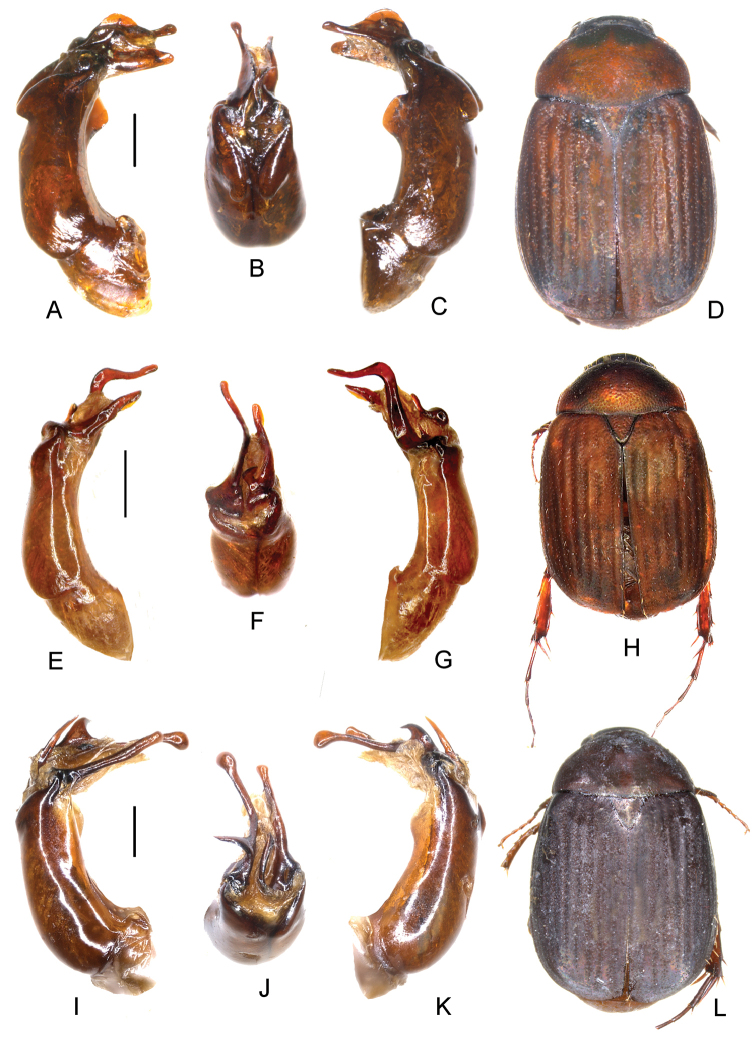
**A–D** *Neoserica hainana* (Brenske) (lectotype) **E–H** *Neoserica shoyungi* Ahrens, Fabrizi & Liu, sp. n. (holotype) **I–L** *Neoserica sakoliana* Ahrens, Fabrizi & Liu, sp. n. (holotype) **A, E** aedeagus, left side lateral view **C, G** aedeagus, right side lateral view **B, F** parameres, dorsal view **D, H** habitus. Scale bars: 0.5 mm. Habitus not to scale.

Aedeagus: Fig. 3A–C. Habitus: Fig. 3D.

##### Variation.

The colour varies from an entirely black body, or a blackish anterior body (head and pronotum) with reddish elytra, to a nearly entirely reddish body with dark head and anterior pronotum, dorsal surface sometimes with greenish shine. Female: pygidium moderately convex, at middle strongly shiny and finely punctate; antennal club slightly shorter than the remaining antennomeres combined, composed of 4 antennomeres.

#### Neoserica (s.l.) shuyongi

Taxon classificationAnimaliaColeopteraScarabaeidae

Ahrens, Fabrizi & Liu sp. n.

http://zoobank.org/B0DCDC55-D503-4263-9013-D51163B33251

Figs 3E–H , 6

##### Type material examined.

Holotype: ♂ “Tianchi, Mt. Jianfengling, Hainan, 25.IV.1980, 750m, leg. Wang Shuyong” (IZAS). Paratypes: 1 ♂ “Mts. Jianfengling, Hainan, 1.IV.1984, leg. Lin Youdong” (IZAS), 1 ♀ “Mts. Jianfengling, Hainan, 10.IV.1980, 800m, leg. Wang Shuyong” (IZAS), 1 ♂ “Tianchi, Mt. Jianfengling, Hainan, 18.IV.1980, 700m, leg. Pu Fuji” (ZFMK).

##### Description.

Body length: 5.9 mm, length of elytra: 3.9 mm, body width: 3.6 mm. Body short-oval, dark brown partly reddish, dorsal surface except anterior labroclypeus moderately dull, pronotum and elytra glabrous.

Labroclypeus subtrapezoidal, distinctly wider than long, widest at base, lateral margins weakly convex, convergent anteriorly; anterior angles strongly rounded; anterior margin shallowly sinuate medially, margins distinctly reflexed; surface nearly flat, shiny including base, coarsely and densely punctate, behind anterior margin with a few even coarser punctures each bearing an erect seta; frontoclypeal suture distinctly incised, weakly curved medially; smooth area in front of eye convex, nearly as long as wide; ocular canthus moderately short and triangular (1/3 of ocular diameter), sparsely punctate, with one terminal seta. Frons with moderately coarse and dense punctures, with a few long erect setae beside eyes and behind frontoclypeal suture. Eyes small, ratio diameter/ interocular width: 0.47. Antenna with nine antennomeres, club (♂) with four antennomeres and straight, 1.2 times as long as remaining antennomeres combined. Mentum convexly elevated and flattened anteriorly.

Pronotum transverse, widest at base, lateral margins evenly convex and moderately convergent anteriorly; anterior angles distinctly produced and sharp, posterior angles blunt and weakly rounded at tip; anterior margin straight, with a fine complete marginal line; surface densely and moderately coarsely punctate, glabrous, with minute setae in punctures (100× magnification); lateral border densely setose; hypomeron distinctly carinate basally, not produced ventrally. Scutellum triangular, with moderately coarse and dense punctures, glabrous.

Elytra short-oval, widest shortly behind middle, striae finely impressed, finely and densely punctate, intervals moderately convex, with sparse, fine punctures concentrated along striae, glabrous except a few short setae on odd intervals; epipleural edge robust, ending at rounded external apical angle of elytra, epipleura densely setose; apical border without a fine fringe of microtrichomes (visible at 100× magnification).

Ventral surface dull, finely and densely punctate; metasternum with a few short setae and long robust setae on metasternal disc; metacoxa glabrous, with a few single setae laterally; abdominal sternites finely and densely punctate, with a transverse row of coarse punctures, each bearing a short robust seta, last sternite half as long as penultimate one. Mesosternum between mesocoxae as wide as the mesofemur, with a semi-circular ridge bearing long setae. Ratio of length of metepisternum/ metacoxa: 1/ 1.72. Pygidium dull, moderately convex, coarsely and densely punctate, without smooth midline, with a few long setae along apical margin.

Legs short; femora dull, with two rudimentary longitudinal rows of setae, finely and sparsely punctate, glabrous; metafemur shiny, with anterior margin acute, without serrated line behind anterior edge, posterior margin apically serrate ventrally, in apical half only weakly widened, posterior margin distinctly serrate dorsally. Metatibia wide and short, widest at middle, ratio of width/ length: 1/ 3.1; dorsal margin only in posterior quarter carinate, otherwise longitudinally convex, with two groups of spines, basal group at one third, apical group at three quarters of metatibial length, basally with a few short single setae; lateral face convex, finely and sparsely punctate, smooth along middle in posterior half; ventral edge finely serrated, with three robust nearly equidistant setae; medial face smooth, apex interiorly near tarsal articulation bluntly truncate and slightly concavely sinuate. Tarsomeres ventrally with sparse, short setae, smooth, neither laterally nor dorsally carinate; metatarsomeres with a strongly serrated ridge ventrally, glabrous; first metatarsomere slightly longer than following two tarsomeres combined and distinctly longer than dorsal tibial spur. Protibia short, bidentate, distal tooth sharply pointed at apex; anterior claws symmetrical, basal tooth of inner claw sharply truncate at apex.

Aedeagus: Fig. 3E–G. Habitus: Fig. 3H.

##### Diagnosis.

*Neoserica shuyongi* Ahrens, Fabrizi & Liu, sp. n. differs from all other species of the *Neoserica obscura* group by the serrate posterior margin of metafemur, antenna composed of 9 antennomeres, shiny base of the clypeus and the shape of the aedeagus (Fig. 3E–G).

##### Etymology.

The new species is named after its collector, Wang Shuyong.

##### Variation.

Among the paratypes no apparent size variation was found; colour varied from entirely reddish brown to dark brown. Female: pygidium less convex, antennal club in the paratype missing.

#### Neoserica (s.l.) sakoliana

Taxon classificationAnimaliaColeopteraScarabaeidae

Ahrens, Fabrizi & Liu sp. n.

http://zoobank.org/D21DD61A-7938-4F0A-9682-E3B101763B2B

Figs 3I–L , 6

##### Type material examined.

Holotype: ♂ “China: Hainan I., No-dong nr. Sa ko lia 12.VII.1935/ L. & M. Gressitt Collectors BISHOP Mus.” (BPBM). Paratypes: 2 ♂♂, 1 ♀ “Mts. Limushan, Qiongzhong, Hainan, 22-23.VII.2006, leg. Wang Jiliang, Gao Chao” (HBUM), 1 ♂ “Xiangshui, Boluo, Guangdong, 30.V.1965, leg. Zhang Youwei” (IZAS), 1 ♂ “Kwangtung, S. China, Tsung Hau, Mei-hsien (District), 19-21.VII.1933, leg. F. K. To” (SYUG), 1 ♂ “Liuwan Forestry Farm, Yulin Insects 0354, 25.V.1981, leg. Huang Xiaoming” (IZAS), 1 ♂ “Mt. Paiyangshan, Guangxi, 27.V.1984, leg. Lu Xiaoshan” (NWAFU), 1 ♂ “Hongchagou, Xishan Forestry Farm, Rong’an, Guangxi, 26.VII.2007, leg. Yang Ganyan” (IZAS), 1 ♂ “Xiangshui, Boluo, Guangdong, 30.V.1965, leg, Zhang Youwei” (IZAS), 1 ♂ “Xinzuochang, Boluo, Guangdong, 3.VI.1965, leg. Zhang Youwei” (IZAS), 1 ♂ “Mt. Diaoluoshan, Hainan, 22.IV.1980, 1000m, leg. Wang Shuyong” (IZAS), 1 v “Wanning, Hainan, Guangdong, 16.IV.1960, 10m, leg. Li Suofu”(ZFMK), 1 ♂ “Sean No.19, Yaosam (Kwangsi), 16.VII.1934, leg. H.C. Tao” (IZAS).

##### Diagnosis.

*Neoserica sakoliana* Ahrens, Fabrizi & Liu, sp. n. is in external appearance and genital morphology similar to *Neoserica allobscura*. *Neoserica sakoliana* differs by the distinctly longer parameres.

##### Description.

Body length: 6.4 mm, length of elytra: 4.4 mm, body width: 4.2 mm. Body short-oval, dark brown, ventral face reddish brown, entire surface except anterior labroclypeus dull, head with some greenish shine, pronotum and elytra glabrous.

Labroclypeus subtrapezoidal, distinctly wider than long, widest at base, lateral margins weakly convex, convergent anteriorly; anterior angles strongly rounded; anterior margin shallowly sinuate medially, margins moderately reflexed; surface weakly convex, shiny, base dull, finely and densely punctate, mixed with a few larger punctures bearing each an erect seta; frontoclypeal suture distinctly incised, weakly curved medially; smooth area in front of eye convex, nearly as long as wide; ocular canthus short and triangular (1/3 of ocular diameter), sparsely punctate, with a terminal seta. Frons with fine and moderately dense punctures, with a few long erect setae beside eyes and behind lateral frontoclypeal suture. Eyes small, ratio diameter/ interocular width: 0.4. Antenna with ten antennomeres, club (♂) with four antennomeres and straight, slightly longer than remaining antennomeres combined. Mentum convexly elevated and flattened anteriorly.

Pronotum transverse, widest at base, lateral margins evenly convex and moderately convergent anteriorly; anterior angles distinctly produced and sharp, posterior angles blunt and weakly rounded at tip; anterior margin straight, with a fine complete marginal line; surface densely and finely punctate, glabrous, with minute setae in punctures (100× magnification); lateral border densely setose; hypomeron distinctly carinate basally, not produced ventrally. Scutellum triangular, with fine, dense punctures, on midline impunctate, glabrous.

Elytra short-oval, widest shortly behind middle, striae finely impressed, finely and densely punctate, intervals weakly convex, with sparse, fine punctures concentrated along striae, except a few robust setae on penultimate external intervals glabrous; epipleural edge robust, ending at nearly blunt external apical angle of elytra, epipleura densely setose; apical border without a fine fringe of microtrichomes (visible at 100× magnification).

Ventral surface dull, finely and densely punctate; metasternum nearly glabrous except a few long robust setae on disc, punctures with minute setae (100× magnification); metacoxa glabrous, with a few single setae laterally; abdominal sternites finely and densely punctate, with a transverse row of coarse punctures, each bearing a short robust seta, last sternite half as long as penultimate one. Mesosternum between mesocoxae as wide as mesofemur, with a semi-circular ridge bearing long setae. Ratio of length of metepisternum/ metacoxa: 1/ 2.2. Pygidium dull, moderately convex, coarsely and densely punctate, without smooth midline, with a few long setae along apical margin.

Legs short; femora moderately shiny, with two rudimentary longitudinal rows of setae, finely and sparsely punctate, glabrous; metafemur with anterior margin acute, without serrated line behind anterior edge, posterior margin smooth ventrally, in apical half only weakly widened, posterior margin smooth dorsally. Metatibia wide and short, widest at middle, ratio of width/ length: 1/ 2.6; dorsal margin sharply carinate, with two groups of spines, basal group at one third, apical group at three quarters of metatibial length, basally with a few short single setae; lateral face weakly convex, finely and sparsely punctate; ventral edge finely serrated, with three robust nearly equidistant setae; medial face smooth, apex interiorly near tarsal articulation bluntly truncate and slightly concavely sinuate. Tarsomeres ventrally with sparse, short setae, smooth, neither laterally nor dorsally carinate; metatarsomeres with a strongly serrated ridge ventrally, glabrous; first metatarsomere slightly shorter than following two tarsomeres combined and as long as dorsal tibial spur. Protibia short, bidentate, distal tooth sharply pointed at apex; anterior claws symmetrical, basal tooth of inner claw sharply truncate at apex.

Aedeagus: Fig. 3I–K. Habitus: Fig. 3L.

##### Etymology.

The name of the new species is derived from the type locality, Sa ko lia.

##### Variation.

Body length: 6.4–8.1 mm, length of elytra: 4.4–5.2 mm, body width: 4.2–5.3 mm. Colour varies from entirely dark reddish brown to nearly black, often with dark pronotum and brown elytra. Female: antennal club composed of 4 antennomeres, first joint of club slightly shorter than the club, club slightly shorter than remaining antennomeres combined.

#### Neoserica (s.l.) tahianensis

Taxon classificationAnimaliaColeopteraScarabaeidae

Ahrens, Fabrizi & Liu sp. n.

http://zoobank.org/3BCE3BC2-46A3-45AB-8408-4422458D3F95

Figs 4A–D , 6

##### Type material examined.

Holotype: ♂ “Hainan I. (C.): Ta Hian (TaSianKwang) 600m. VI-10-35 J.L. Gressitt” (BPBM). Paratypes: 1 ♂, 1 ♀ “Yinggen, Hainan, Guangdong, 10.VII.1960, 200m, leg. Zhang Xuezhong” (IZAS), 1 ♂ “Tongshi, Hainan, Guangdong, 31.VII.1960, 340m, leg. Li Suofu” (IZAS), 3 ♂♂, 3 ♀♀ “Shuiman, Hainan, Guangdong, 29.V.1960, 640m, leg. Zhang Xuezhong” (IZAS, ZFMK), 1 ♂ “Tongshi, Hainan, Guangdong, 6.VIII.1960, 340m, leg. Li Suofu” (IZAS).

##### Diagnosis.

*Neoserica tahianensis* Ahrens, Fabrizi & Liu, sp. n. is in external appearance and genital morphology similar to *Neoserica obscura* and *Neoserica allobscura*. *Neoserica tahianensis* differs by the large ventral process of the phallobase and by the shape of its parameres: the right paramere is slightly longer than in *Neoserica obscura*, the left one does not possess a dorsal lobe.

##### Description.

Body length: 6.5 mm, length of elytra: 4.3 mm, body width: 4.3 mm. Body short-oval, dark brown, elytra black, abdomen dark brown, dorsal surface except anterior labroclypeus dull, head and pronotum with some greenish shine, pronotum and elytra glabrous.

Labroclypeus subtrapezoidal, distinctly wider than long, widest at base, lateral margins weakly convex, convergent anteriorly; anterior angles strongly rounded; anterior margin shallowly sinuate medially, margins moderately reflexed; surface weakly convex, shiny, base dull, finely and densely punctate, mixed with a few larger punctures bearing each an erect seta; frontoclypeal suture distinctly incised, weakly curved medially; smooth area in front of eye convex, nearly as long as wide; ocular canthus short and triangular (1/3 of ocular diameter), sparsely punctate, without terminal seta. Frons with fine and moderately dense punctures, with a few long erect setae beside eyes and behind lateral frontoclypeal suture. Eyes small, ratio diameter/ interocular width: 0.42. Antenna with ten antennomeres, club (♂) with four antennomeres and straight, slightly longer than remaining antennomeres combined. Mentum convexly elevated and flattened anteriorly.

Pronotum transverse, widest at base, lateral margins evenly convex and moderately convergent anteriorly; anterior angles distinctly produced and sharp, posterior angles blunt and weakly rounded at tip; anterior margin straight, with a fine complete marginal line; surface densely and finely punctate, glabrous, with minute setae in punctures (100× magnification); lateral border densely setose; hypomeron distinctly carinate basally, not produced ventrally. Scutellum triangular, with fine, dense punctures, glabrous.

Elytra short-oval, widest shortly behind middle, striae finely impressed, finely and densely punctate, intervals weakly convex, with sparse, fine punctures concentrated along striae, except a few robust setae on sutural interval glabrous; epipleural edge robust, ending at nearly blunt external apical angle of elytra, epipleura densely setose; apical border without a fine fringe of microtrichomes (visible at 100× magnification).

Ventral surface dull, finely and densely punctate; metasternum nearly glabrous except a few long robust setae on disc, punctures with minute setae (100× magnification); metacoxa glabrous, with a few single setae laterally; abdominal sternites finely and densely punctate, with a transverse row of coarse punctures, each bearing a short robust seta, last sternite half as long as penultimate one. Mesosternum between mesocoxae as wide as the mesofemur, with a semi-circular ridge bearing long setae. Ratio of length of metepisternum/ metacoxa: 1/ 2.2. Pygidium dull, moderately convex, coarsely and densely punctate, without smooth midline, with a few long setae along apical margin.

Legs short; femora moderately shiny, with two rudimentary longitudinal rows of setae, finely and sparsely punctate, glabrous; metafemur with anterior margin acute, without serrated line behind anterior edge, posterior margin smooth ventrally, in apical half only weakly widened, posterior margin smooth dorsally. Metatibia wide and short, widest at middle, ratio of width/ length: 1/ 2.7; dorsal margin sharply carinate, with two groups of spines, basal group at one third, apical group at three quarters of metatibial length, basally with a few short single setae; lateral face weakly convex, finely and sparsely punctate; ventral edge finely serrated, with three robust nearly equidistant setae; medial face smooth, apex interiorly near tarsal articulation bluntly truncate and slightly concavely sinuate. Tarsomeres ventrally with sparse, short setae, smooth, neither laterally nor dorsally carinate; metatarsomeres with a strongly serrated ridge ventrally, glabrous; first metatarsomere slightly shorter than following two tarsomeres combined and slightly shorter than dorsal tibial spur. Protibia short, bidentate, distal tooth sharply pointed at apex; anterior claws symmetrical, basal tooth of inner claw sharply truncate at apex.

**Figure 4.**
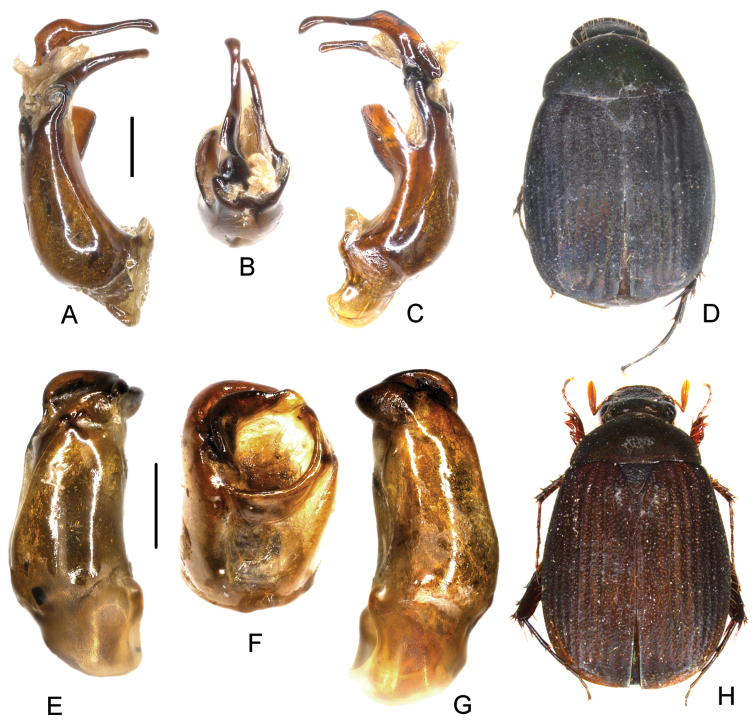
**A–D** *Neoserica tahianensis* Ahrens, Fabrizi & Liu, sp. n. (holotype) **E–H** *Neoserica silvestris* Brenske (China: Nu Shan) **A, E** aedeagus, left side lateral view **C, G** aedeagus, right side lateral view **B, F** parameres, dorsal view **D, H** habitus. Scale bars: 0.5 mm. Habitus not to scale.

Aedeagus: Fig. 4A–C. Habitus: Fig. 4D.

##### Etymology.

The name of the new species is derived from the type locality, Ta Hian.

##### Variation.

Body length: 6.5–7.1 mm, length of elytra: 4.3–4.6 mm, body width: 4.3–4.7 mm. Colour varies from entirely reddish brown to nearly completely black, often reddish elytra and pronotum with a dark margin. Female: Antennal club composed of 4 antennomeres, first joint of club slightly shorter than the club, club slightly shorter than remaining antennomeres combined.

### *Neoserica silvestris* group

**Diagnosis.** Body moderately small (7–8 mm), oval, moderately convex; unicoloured black or reddish-brown, dorsal surface dull or with some iridescent shine, nearly glabrous. Antenna with 10 antennomeres, dark; antennal club of ♂ composed of 4 antennomeres, in ♀ of 3 antennomeres, but club shorter than remaining antennomeres combined. Hypomeron basally carinate. Protibia bidentate. Metatibia at apex moderately sinuate close to tarsal articulation. Metafemur with serrated line adjacent to anterior margin. Metatibia moderately wide, with serrated longitudinal line in basal half.

**Remarks.** The species group is based on *Neoserica silvestris* Brenske, 1902, and proposed here to accommodate the species closely related to *Neoserica silvestris*.

**Distribution.** So far only known from China and northern Myanmar.

#### Key to the Chinese species of the *Neoserica silvestris* group:

**Table:** 

1	Labroclypeus with a distinct transverse elevation. Antennal club only little longer than the remaining antennomeres combined.	***Neoserica silvestris***
–	Labroclypeus flat. Antennal club more than 1.5 times as long as the remaining antennomeres combined.	**2**
2	Left paramere shorter and less widely sinuated, its tip is directed straight forward	***Neoserica minor***
–	Left paramere longer and more widely sinuated, its tip is not straight but curved interiorly.	***Neoserica pseudosilvestris* Ahrens, Fabrizi & Liu sp. n.**

#### Neoserica (s.l.) silvestris

Taxon classificationAnimaliaColeopteraScarabaeidae

Brenske, 1902

Figs 4E–H , 6

Neoserica silvestris Brenske, 1902: 61.

##### Type material examined.

Syntypes: 2 ♂♂ “Ho-chan/ coll. Thery” (BMNH), 1 ♀ “China Ho-chan/ *Serica silvestris* typ. Brsk./ coll. Brenske” (ZMHB).

##### Additional material examined.

**CHINA**: 1 ex. “China: (Yunnan) Nujiang Lisu Aut. Pref., Nu Shan, 7 km NNW Caojian, 2420m, 25°43'29"N 99°07'57"E (shrubs, litter, moss shifted) 11.VI.2007 leg. D. Wrase/ DA1553” (ZFMK), 4 ex. “China Sichuan Moxi, VI.1993 M. Hackel lgt.” (CN), 1 ex. (♀) “China: Sichuan; Moxi; 29.13N 102.10E, 1600m, 2.vii.1998/ 1998 China Expedition J. Farkac, D. Kral, A. Smetana & J. Schneider” (CP), 8 ex. “Sichuan 1950 m Luding, Xin Shing 1.VI.1990 A. Vigna leg.” (CS), 1 ex. “Sichuan, Moxi Gongashan Mts. 28.VI.-2.VII.1994 Bolm lgt. 1650 m” (CP), 1 ex. “China, E Hubei, 7-10.V. Dabie Shan, 31.1N 115.8E Wujiashan forest park Jaroslav Turna leg., 2004” (ZFMK), 3 ♂♂ “China: Sichuan; Wolong Reserve, Sigulian Shan, 31°09'N 103°06'E v.2006, 1500-1800m V. Siniaev” (ZFMK), 12 ex. “Yunnan 2900-3500m 27.01N 100.12E 1993 Yulongshan mts. 24-26/5. Vit Kuban leg.” (CP), 5 ex. “Yunnan 2200-2500m 24.57N 98.45E 8-16/5 Gaoligong mts. Vit Kuban leg. 1995” (CP), 2 ex. “C-China, Shaanxi, Qinling Shan, 6km E of Xunyangba 1000-1300m, 23.V.-13.VI. Leg. C. Holzschuh 2000” (CP), 6 ex. “C China, W Sichuan, Luding Xian, Moxi, 9-14.vii.1999, V. Benes leg.” (CP), 1 ex. “China-Shaanxi, Daba Shan, Shou Man vil., 32°14'N, 108°34'E, 25.v.-14.vi.2000, 1000m, Siniaev & Plutenko leg.” (CP), 1 ex. “China: Yunnan prov., Gaoligongshan mts.; 90km W of Baoshan; S. Becvar leg.; 26.-29.v.1995” (CP), 2 ex. “China, 1000-1300m, Shaanxi, Qinling mts., Xunyangba (6km E) 23,v.-13.vi.1998, J.H. Mashal leg. (CP), 1 ex. (♀) “China, Daxue Shan Mts., Sichuan, Gongga Shan Mt., Moxi, 11-13.vii.1999, 1700m, 29°39'N, 102°06'E, V. Siniaev & A. Plutenko lgt.” (CP), 1 ex. “China: N-Yunnan Baiyungshan (Bai Railing Mts.) 2400 m Yong Ren, VII-2003 leg. Ying et al.” (ZFMK), 1 ♂ “China: Hunan; Mupu Mt. 1600m, Pingjiang VIII-2003, leg. Li et al.” (ZFMK), 2 ♀♀ “China West Sichuan Moximian Luding Co. 13.-18.7.94 Benes” (ZFMK), 2 ex. (♀) “Yunnan 2000-3000m 25.11N 100.24E Weibaoshan mts. W slope 25-28/6.92 Vit Kuban leg.” (ZFMK), 1 ex. “Den Shiang Uen nr Ningyuenfu/ viii. 9-10-’28 8000-9500ft./ China DC Graham” (USNM), 1 ex. Chengtu 1933/ Szechwan China DC Graham XI-28 alt. 1700ft.” (USNM), 1 ex. “Yachow dist. May ‘28 Coll’r Chen Gih Uen/ Szechuen China DC Graham” (USNM), 1 ex. “Kuanshan Szechwan China DC Graham 19-20-33 alt. 2200-5200ft.” (USNM), 1 ♂, 2 ♀♀ “Heilongtan, Kunming, Yunnan, 5.IV.1956, 1900m, leg. Huang Keren *etc.*” (IZAS), 1 ♂ “Heilongtan, Kunming, Yunnan, 5.IV.1956, 1900m, leg. Huang Keren” (IZAS), 1 ♂ “Lomgmenhe, Xingshan, Hubei, 21.VI.1993, 1260m, leg. Li Hongxing” (IZAS), 1 ♂ “Kunming, Yunnan, 7.VI.1955, 1900m, leg. Li Xiwen” (IZAS), 1 ♂ “Menghai, Xishuangbanna, Yunnan, 18.VII.1958, 1200-1600m, leg. Wang Shuyong” (IZAS), 1 ♂ “Institute of Agricultural Sciences, Bijie, Guizhou, leg. Yang, No. 55” (IZAS), 1 ♂ “Botany Garden, Guiyang, Guizhou, 1981” (IZAS), 1 ♂ “Louguantai, Qinling, 30.V.1951” (NWAFU), 1 ♂ “Mt. Taishan, Shangdong, 31.V.1956” (IZAS), 1 ♂ “Xinxing, Luding, Sichuan, 15.VI.1983, 1900m, leg. Chen Yuanqing” (IZAS), 1 ♂ “Yunlong, Yunnan, 20.VI.1981, 2450m, leg. Liao Subai” (IZAS), 1 ♂ “Mts. Lushan, Yiyuan, Shandong, 19.V.2007, leg. Wang Fengyan, Wang Jiliang, Wu Qiqi” (HBUM). **MYANMAR**: 2 ♂♂ “Myanmar (Burma) Provinz Kachin State Kanphat/ Grenze zu China 24.V.2006 leg. Michael Langer, Stefan Naumann & Swen Loeffler coll. M. Langer/ Nachtfang/ 1642m N26°08'512" E098°34'582" “ (ZFMK), 1 ♂ “Myanmar (Burma) Provinz Kachin State ca 20 km N von Panwar 23.V.2006 leg. Michael Langer, Stefan Naumann & Swen Loeffler coll. M. Langer/ Nachtfang/ 2180m N25°43'302" E098°23'353" “ (ZFMK).

##### Redescription.

Body length: 8.1 mm, length of elytra: 4.0 mm, body width: 3.8 mm. Body short-oval, black to dark brown, antenna yellow, dorsal surface except labroclypeus dull or with iridescent or greenish shine, pronotum and elytra glabrous.

Labroclypeus subtrapezoidal, distinctly wider than long, widest at base, lateral margins strongly convex, convergent anteriorly; anterior angles strongly rounded; anterior margin distinctly sinuate medially, margins moderately reflexed; surface with a convex transverse ridge, moderately shiny, coarsely and very densely punctate, with a few erect setae anteriorly; frontoclypeal suture finely incised, evenly curved; smooth area in front of eye convex, 1.5 times as wide as long; ocular canthus short and triangular (1/3 of ocular diameter), densely and finely punctate, with one terminal seta. Frons with fine and sparse punctures, with two long erect setae beside eyes. Eyes small, ratio diameter/ interocular width: 0.51. Antenna with ten antennomeres, club (♂) with four antennomeres and straight, slightly longer than remaining antennomeres combined. Mentum convexly elevated and flattened anteriorly.

Pronotum transverse, widest at base, lateral margins evenly convex and weakly convergent anteriorly; anterior angles distinctly produced and sharp, posterior angles blunt and moderately rounded at tip; anterior margin convex, with a very fine but complete marginal line; surface densely and finely punctate, glabrous, with minute setae in punctures (100× magnification); lateral border densely setose; hypomeron distinctly carinate basally, not produced ventrally. Scutellum triangular, with fine, dense punctures, glabrous.

Elytra oval, widest in posterior third, striae finely impressed, finely and densely punctate, intervals weakly convex, with dense, fine punctures concentrated along striae, glabrous except a few single, short setae on penultimate lateral interval; epipleural edge robust, ending at convex external apical angle of elytra, epipleura densely setose; apical border with a fine fringe of microtrichomes (visible at 100× magnification).

Ventral surface dull, finely and densely punctate; metasternum nearly glabrous except a few long robust setae on disc, punctures with minute setae (100× magnification); metacoxa glabrous, with a few single setae laterally; abdominal sternites finely and densely punctate, with a transverse row of coarse punctures, each bearing a short robust seta, last sternite half as long as penultimate one. Mesosternum between mesocoxae as wide as the mesofemur, with a semi-circular ridge bearing long setae. Ratio of length of metepisternum/ metacoxa: 1/ 1.49. Pygidium dull, strongly convex, finely and densely punctate, without smooth midline, with a few long setae along apical margin.

Legs short; femora moderately shiny, with two longitudinal rows of setae, finely and sparsely punctate, glabrous; metafemur with anterior margin acute, with a continuous, serrated line behind anterior edge, posterior margin smooth ventrally, in apical half only weakly widened, posterior margin smooth dorsally. Metatibia moderately wide and short, widest at middle, ratio of width/ length: 1/ 2.95; dorsal margin sharply carinate, with two groups of spines, basal group shortly behind middle, apical group at 4/5 of metatibial length, in basal half with a serrated line beside dorsal margin ending at basal group of spines, beside it with a few single short setae; lateral face longitudinally convex, finely and densely punctate, smooth along middle; ventral edge finely serrated, with three robust nearly equidistant setae; medial face smooth, apex interiorly near tarsal articulation bluntly truncate and slightly concavely sinuate. Tarsomeres sparsely and finely punctate dorsally, with sparse, short setae ventrally, neither laterally nor dorsally carinate; metatarsomeres with a strongly serrated ridge ventrally, glabrous; first metatarsomere slightly shorter than following two tarsomeres combined and one third of its length longer than dorsal tibial spur. Protibia short, bidentate, distal tooth sharply pointed at apex, external margin bluntly widened at middle; anterior claws symmetrical, basal tooth of inner claw sharply truncate at apex.

Aedeagus: Fig. 4E–G. Habitus: Fig. 4H.

##### Variation.

The colour varies from totally black or reddish brown to black with reddish brown elytra. Female: Antennal club also composed of 3 antennomeres, however, the club is slightly shorter than in males, and the first joint of club is slightly shorter.

#### Neoserica (s.l.) minor

Taxon classificationAnimaliaColeopteraScarabaeidae

(Arrow, 1946) comb. n.

Figs 5A–D , 6

Aserica minor Arrow, 1946a: 15.

##### Type material examined.

Syntype: ♂ “N. E. Burma Kambaiti 7000 ft. 24/5.1934/ N. E. Burma R. Malaise B. M. 1945-71/Co-Type/ Aserica minor Arrow co-type” (BMNH).

##### Additional material examined.

1 ♂ “China, W Yunnan prov., mts. 60km E Tengchong, 2200m, 19.-22.v.2006, S. Murzin & I. Shokhin” (CP).

##### Redescription.

Body length: 7.2 mm, length of elytra: 5.2 mm, body width: 4.4 mm. Body short-oval, black to dark brown, antenna yellow, dorsal surface except labroclypeus dull, pronotum and elytra glabrous.

Labroclypeus subtrapezoidal, distinctly wider than long, widest at base, lateral margins weakly convex, convergent anteriorly; anterior angles moderately rounded; anterior margin distinctly sinuate medially, margins moderately reflexed; surface nearly flat, moderately shiny, coarsely and very densely punctate, with a few erect setae anteriorly; frontoclypeal suture finely incised, convexly bent at middle; smooth area in front of eye convex, 1.2 times as wide as long; ocular canthus short and triangular (1/3 of ocular diameter), densely and finely punctate, with one terminal seta. Frons with coarse and dense punctures, glabrous. Eyes small, ratio diameter/ interocular width: 0.51. Antenna with ten antennomeres, club (♂) with four antennomeres and straight, 1.7 times as long as remaining antennomeres combined. Mentum convexly elevated and flattened anteriorly.

Pronotum transverse, widest at base, lateral margins in basal half nearly straight and moderately convergent, in anterior half evenly convex and weakly convergent anteriorly; anterior angles distinctly produced and sharp, posterior angles blunt and moderately rounded at tip; anterior margin convex, with a very fine but complete marginal line; surface densely and finely punctate, glabrous, with minute setae in punctures (100× magnification); lateral border densely setose; hypomeron distinctly carinate basally, not produced ventrally. Scutellum triangular, with fine, dense punctures, glabrous.

Elytra oval, widest in posterior third, striae finely impressed, finely and densely punctate, intervals weakly convex, with dense, fine punctures concentrated along striae, glabrous except a few single, short setae on penultimate lateral interval; epipleural edge robust, ending at convex external apical angle of elytra, epipleura densely setose; apical border with a fine fringe of microtrichomes (visible at 100× magnification).

Ventral surface dull, finely and densely punctate; metasternum nearly glabrous except a few long robust setae on disc, punctures with minute setae (100× magnification); metacoxa glabrous, with a few single setae laterally; abdominal sternites finely and densely punctate, with a transverse row of coarse punctures, each bearing a short robust seta, last sternite half as long as penultimate one. Mesosternum between mesocoxae as wide as the mesofemur, with a semi-circular ridge bearing long setae. Ratio of length of metepisternum/ metacoxa: 1/ 1.52. Pygidium dull, strongly convex, finely and densely punctate, without smooth midline, with a few long setae along apical margin.

Legs moderately long; femora moderately shiny, with two longitudinal rows of setae, finely and sparsely punctate, glabrous; metafemur with anterior margin acute, with a continuous, serrated line behind anterior edge, posterior margin smooth ventrally, in apical half only weakly widened, posterior margin smooth dorsally. Metatibia narrow and moderately long, widest at middle, ratio of width/ length: 1/ 3.35; dorsal margin sharply carinate, with two groups of spines, basal group shortly behind middle, apical group at 4/5 of metatibial length, in basal half with a serrated line beside dorsal margin ending at basal group of spines, beside it with a few single short setae; lateral face longitudinally convex, finely and densely punctate, smooth along middle; ventral edge finely serrated, with three robust nearly equidistant setae; medial face smooth, apex interiorly near tarsal articulation bluntly truncate and slightly concavely sinuate. Tarsomeres sparsely and finely punctate dorsally, with sparse, short setae ventrally, neither laterally nor dorsally carinate; metatarsomeres with a strongly serrated ridge ventrally, glabrous; first metatarsomere slightly shorter than following two tarsomeres combined and one third of its length longer than dorsal tibial spur. Protibia short, bidentate, distal tooth sharply pointed at apex, external margin bluntly widened at middle; anterior claws symmetrical, basal tooth of inner claw sharply truncate at apex.

**Figure 5.**
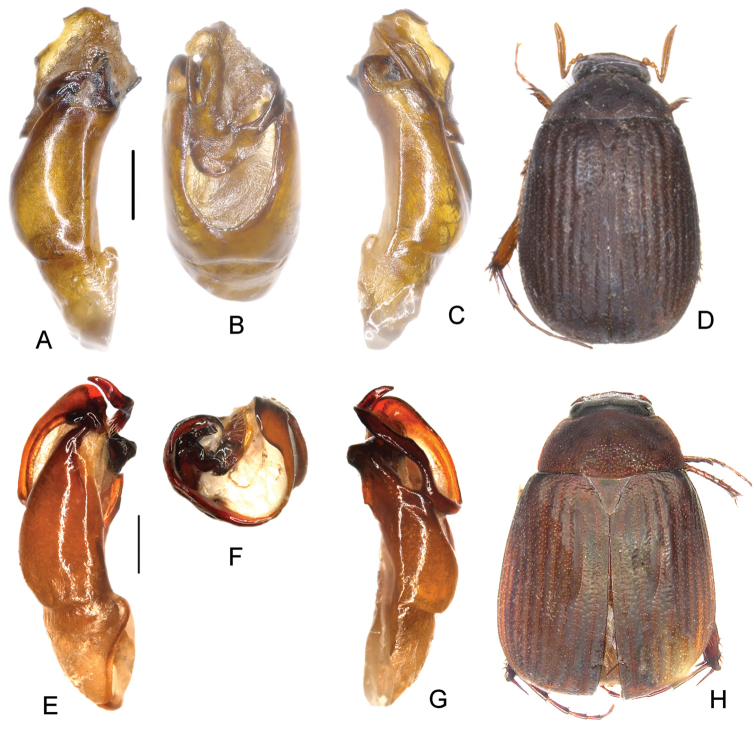
**A–D** *Neoserica minor* (Arrow) (China: 60km E Tengchong), **E–H** *Neoserica pseudosilvestris* Ahrens, Fabrizi & Liu, sp. n. (holotype). **A, E** aedeagus, left side lateral view **C, G** aedeagus, right side lateral view **B, F** parameres, dorsal view **D, H** habitus. Scale bars: 0.5 mm. Habitus not to scale.

Aedeagus: Fig. 5A–C. Habitus: Fig. 5D. Female unknown.

#### Neoserica (s.l.) pseudosilvestris

Taxon classificationAnimaliaColeopteraScarabaeidae

Ahrens, Fabrizi & Liu sp. n.

http://zoobank.org/E594F234-FBCF-4DB3-9404-F7D3BAED567F

Figs 5E–H , 6

##### Type material examined.

Holotype: ♂ “[China] Yunnan, Yakou, 2012-V-11/ LW-1319” (IZAS).

##### Diagnosis.

*Neoserica pseudosilvestris* Ahrens, Fabrizi & Liu, sp. n. is in external appearance very similar to *Neoserica minor* (Arrow). The new species differs by the longer left paramere being more widely sinuated and having the tip not straight but curved interiorly.

##### Description.

Body length: 8.0 mm, length of elytra: 6.1 mm, body width: 5.1 mm. Body short-oval, black to dark brown, antenna yellow, dorsal surface except labroclypeus dull, pronotum and elytra glabrous.

Labroclypeus subtrapezoidal, distinctly wider than long, widest at base, lateral margins strongly convex, convergent anteriorly; anterior angles moderately rounded; anterior margin distinctly sinuate medially, margins moderately reflexed; surface nearly flat, shiny, finely and very densely punctate, with a few erect setae anteriorly; frontoclypeal suture finely incised, convexly bent at middle; smooth area in front of eye convex, 1.3 times as wide as long; ocular canthus long and subtriangular (nearly half of ocular diameter), densely and finely punctate, with one terminal seta. Frons with moderately coarse and dense punctures, with two single erect setae beside eyes. Eyes small, ratio diameter/ interocular width: 0.54. Antenna with ten antennomeres, club (♂) with four antennomeres and straight, 1.7 times as long as remaining antennomeres combined. Mentum convexly elevated and flattened anteriorly.

Pronotum transverse, widest at base, lateral margins evenly convex and weakly convergent anteriorly; anterior angles distinctly produced and sharp, posterior angles blunt and moderately rounded at tip; anterior margin convex, with a very fine but complete marginal line; surface densely and finely punctate, with minute setae in punctures (100× magnification); lateral border densely setose; hypomeron distinctly carinate basally, not produced ventrally. Scutellum triangular, with fine, dense punctures, punctures on basal midline less dense, with minute setae in punctures.

Elytra oval, widest in posterior third, striae finely impressed, finely and densely punctate, intervals weakly convex, with dense, fine punctures concentrated along striae, glabrous except a few single, short setae on penultimate lateral interval; epipleural edge robust, ending at convex external apical angle of elytra, epipleura densely setose; apical border with a fine fringe of microtrichomes (visible at 100× magnification).

Ventral surface dull, finely and densely punctate; metasternum nearly glabrous except a few long robust setae on disc, punctures with minute setae (100× magnification); metacoxa glabrous, with a few single setae laterally; abdominal sternites finely and densely punctate, with a transverse row of coarse punctures, each bearing a short robust seta, last sternite half as long as penultimate one. Mesosternum between mesocoxae as wide as the mesofemur, with a semi-circular ridge bearing long setae. Ratio of length of metepisternum/ metacoxa: 1/ 1.5. Pygidium dull, strongly convex, coarsely and densely punctate, without smooth midline, with a few long setae on apical quarter, otherwise punctures with minute setae.

Legs moderately long; femora moderately shiny, with two longitudinal rows of setae, finely and sparsely punctate, glabrous; metafemur with anterior margin acute, with a continuous, serrated line behind anterior edge, posterior margin smooth ventrally, in apical half only weakly widened, posterior margin smooth dorsally. Metatibia narrow and moderately long, widest at middle, ratio of width/ length: 1/ 3.75; dorsal margin sharply carinate, with two groups of spines, basal group at 3/5, apical group at 4/5 of metatibial length, in basal half with a serrated line beside dorsal margin ending at basal group of spines, beside it with a few single short setae; lateral face longitudinally convex, finely and densely punctate, smooth along middle; ventral edge finely serrated, with three robust nearly equidistant setae; medial face smooth, apex interiorly near tarsal articulation bluntly truncate and slightly concavely sinuate. Tarsomeres sparsely and finely punctate dorsally, with sparse, short setae ventrally, neither laterally nor dorsally carinate; metatarsomeres with a strongly serrated ridge ventrally, glabrous; first metatarsomere slightly shorter than following two tarsomeres combined and one third of its length longer than dorsal tibial spur. Protibia short, bidentate, distal tooth sharply pointed at apex, external margin bluntly widened at middle; anterior claws symmetrical, basal tooth of inner claw sharply truncate at apex.

Aedeagus: Fig. 5E–G. Habitus: Fig. 5H. Female unknown.

##### Etymology.

The name of the new species is the combined Greek prefix “pseudo-” (false) and the species name “*silvestris*” (with reference to the resemblance to *Neoserica silvestris*).

**Figure 6.**
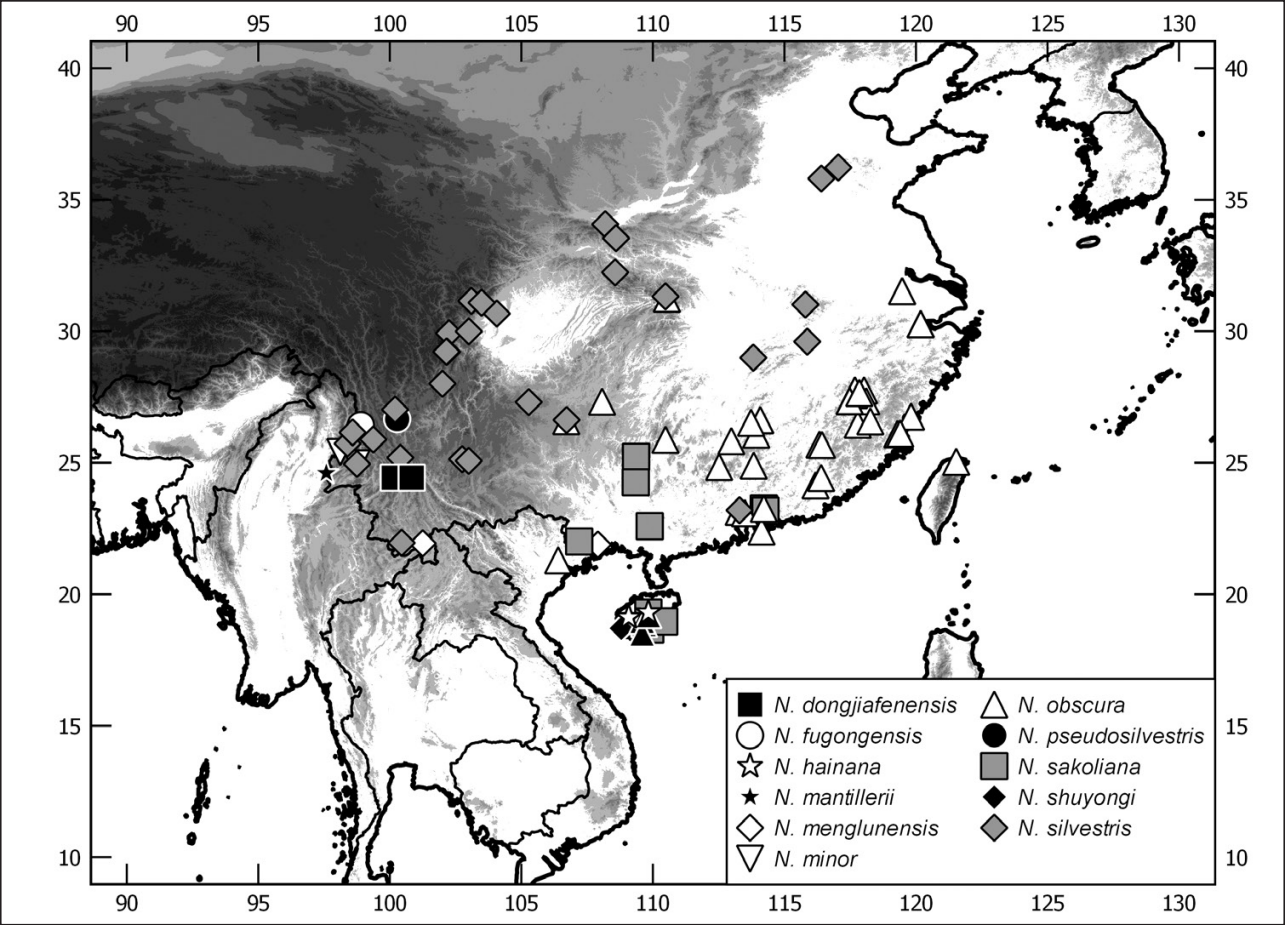
Distribution of the species of the *Neoserica obscura*, *Neoserica lubrica* and *Neoserica silvestris* groups [in China].

## Supplementary Material

XML Treatment for Neoserica (s.l.) fugongensis

XML Treatment for Neoserica (s.l.) mantillerii

XML Treatment for Neoserica (s.l.) dongjiafenensis

XML Treatment for Neoserica (s.l.) menglunensis

XML Treatment for Neoserica (s.l.) obscura

XML Treatment for Neoserica (s.l.) allobscura

XML Treatment for Neoserica (s.l.) hainana

XML Treatment for Neoserica (s.l.) shuyongi

XML Treatment for Neoserica (s.l.) sakoliana

XML Treatment for Neoserica (s.l.) tahianensis

XML Treatment for Neoserica (s.l.) silvestris

XML Treatment for Neoserica (s.l.) minor

XML Treatment for Neoserica (s.l.) pseudosilvestris
